# EGF Relays Signals to COP1 and Facilitates FOXO4 Degradation to Promote Tumorigenesis

**DOI:** 10.1002/advs.202000681

**Published:** 2020-09-23

**Authors:** Hyun Ho Choi, Shaomin Zou, Jian‐lin Wu, Huashe Wang, Liem Phan, Kai Li, Peng Zhang, Daici Chen, Qingxin Liu, Baifu Qin, Thu Anh Thai Nguyen, Sai‐Ching J. Yeung, Lekun Fang, Mong‐Hong Lee

**Affiliations:** ^1^ Guangdong Provincial Key Laboratory of Colorectal and Pelvic Floor Disease The Sixth Affiliated Hospital Sun Yat‐sen University Guangzhou 510655 China; ^2^ Guangdong Research Institute of Gastroenterology The Sixth Affiliated Hospital Sun Yat‐sen University Guangzhou 510655 China; ^3^ State Key Laboratory of Quality Research in Chinese Medicine Macau Institute for Applied Research in Medicine and Health Macau University of Science and Technology Macao 999078 China; ^4^ Department of Colorectal Surgery The Sixth Affiliated Hospital Sun Yat‐sen University Guangzhou 510655 China; ^5^ Department of Molecular and Cellular Oncology Division of Basic Science Research The University of Texas MD Anderson Cancer Center Houston TX 77030 USA; ^6^ Department of Biotechnology Nong Lam University Ho Chi Minh City 700000 Vietnam; ^7^ Department of Emergency Medicine Division of Internal Medicine The University of Texas MD Anderson Cancer Center Houston TX 77030 USA

**Keywords:** COP1, COP9, CSN, FOXO4, SGOC

## Abstract

Forkhead‐Box Class O 4 (FOXO4) is involved in critical biological functions, but its response to EGF‐PKB/Akt signal regulation is not well characterized. Here, it is reported that FOXO4 levels are downregulated in response to EGF treatment, with concurrent elevation of COP9 Signalosome subunit 6 (CSN6) and E3 ubiquitin ligase constitutive photomorphogenic 1 (COP1) levels. Mechanistic studies show that CSN6 binds and regulates FOXO4 stability through enhancing the E3 ligase activity of COP1, and that COP1 directly interacts with FOXO4 through a VP motif on FOXO4 and accelerates the ubiquitin‐mediated degradation of FOXO4. Metabolomic studies demonstrate that CSN6 expression leads to serine and glycine production. It is shown that FOXO4 directly binds and suppresses the promoters of serine‐glycine‐one‐carbon (SGOC) pathway genes, thereby diminishing SGOC metabolism. Evidence shows that CSN6 can regulate FOXO4‐mediated SGOC gene expression. Thus, these data suggest a link of CSN6‐FOXO4 axis and ser/gly metabolism. Further, it is shown that CSN6‐COP1‐FOXO4 axis is deregulated in cancer and that the protein expression levels of CSN6 and FOXO4 can serve as prognostic markers for cancers. The results illustrate a pathway regulation of FOXO4‐mediated serine/glycine metabolism through the function of CSN6‐COP1 axis. Insights into this pathway may be strategically designed for therapeutic intervention in cancers.

## Graphical abstract

Forkhead‐Box Class O 4 (FOXO4) levels are downregulated in response to epidermal growth factor (EGF) treatment, with concurrent elevation of COP9 signalosome subunit 6 (CSN6) and E3 ubiquitin ligase constitutive photomorphogenic 1 levels. EGF‐mediated downregulation of FOXO4 rewires cancer metabolic programming via enhancing the expression of serine‐glycine‐one‐carbon genes. Studies demonstrate that CSN6 and FOXO4 can serve as prognostic markers.

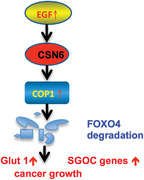

## 1 Introduction

Colorectal cancer (CRC) is accumulated with genetic alterations during the progression and invasion.^[^ ^1^ ^]^ CRC is the third leading malignance in the world population, causing near 500 000 death per year, and its incidence has been a health care challenge.^[^ ^2^ ^]^ CRC continues to be one of the deadliest cancer types with different molecular phenotypes/strong resistance to therapies^[^ ^3^ ^]^ and a very high mortality rate.^[^ ^4^ ^]^ Pathological studies of CRC cancer are focusing on oncogenes, tumor suppressor genes, and even microbiome,^[^ ^5^, ^6^ ^]^ and fungi.^[^ ^7^ ^]^ However, mechanistic studies regarding these aspects remain not well characterized. Therefore, identifying more molecular biomarkers for CRC on the basis of mechanistic studies is an urgent need.

Human Forkhead‐Box Class O (FOXO) transcription factors are critical regulators involved in response to external stimuli, including growth factors, insulin, metabolism, and oxidative stress to control gene‐expression programs.^[^ ^8^ ^]^ There are four members in the FOXO family, including FOXO1, FOXO3A, FOXO4, and FOXO6.^[^ ^9^ ^]^ These members have distinct but overlapping biological functions. For instance, *FOXO1* gene is fused to *PAX3* or *PAX7* genes in rhabdomyosarcoma, and *FOXO3* or *FOXO4* gene is fused with *MLL* gene, thereby causing hematological malignancies.^[^ ^10^ ^]^ Also constitutively active FOXO1 or FOXO3a inhibits endothelial cell migration and tube formation in vitro, but FOXO4 cannot do so.^[^ ^11^ ^]^ Here, we focus on FOXO4, a member deregulated in many types of cancer. It could suppress tumor development through inhibiting cancer cell proliferation (targeting p27, p21), promoting cancer cells apoptosis (targeting Bcl6, caspase3), and hindering cancer cells metastasis (targeting E‐cadherin) and tumor angiogenesis (targeting HIF‐1*α*).^[^ ^12^ ^]^ Its mechanistic role as a tumor suppressor is very important, but the upstream regulators/downstream targets of FOXO4 and its post‐transcriptional modification in tumorigenesis remain not well characterized.

COP9 (Constitutively photomorphogenic 9) signalosome plays a critical role in regulating the degradation of tumor suppressor and oncogene products via ubiquitination and proteasome‐mediated protein degradation. COP9 signalosome subunit 6 (CSN6) is one of the eight subunits of the COP9 signalosome and is involved in ubiquitination,^[^ ^13^, ^14^ ^]^ cell cycle,^[^ ^15^, ^16^ ^]^ transcriptional activation,^[^ ^17^ ^]^ signal transduction,^[^ ^14^, ^16^ ^]^ and tumorigenesis.^[^ ^14^, ^18^ ^]^ Other biological functions of CSN6 remain to be explored.

CSN6 targets several important E3 ligases such as MDM2 and *β*‐trcp to have biological impacts.^[^ ^13^, ^19^ ^]^ Mammalian COP1 (constitutive photomorphogenic 1) is an E3 ligase that is ubiquitously expressed. COP1 overexpression is observed in many types of cancer. Originally characterized in plant, COP1 has a pivotal role in light signaling in plants, but its role in mammals is much more complex. Mammalian COP1 functions as an E3 ubiquitin ligase targeting several substrates, including c‐Jun,^[^ ^20^ ^]^ ETV1,^[^ ^21^ ^]^ p53,^[^ ^22^ ^]^ acetyl‐CoA carboxylase,^[^ ^23^ ^]^ TORC2,^[^ ^24^ ^]^ and MTA1.^[^ ^25^, ^26^ ^]^ COP1 acts as an oncoprotein in tumorigenesis as it can suppress tumor suppressors: p53, p27, and 14‐3‐3 sigma activity,^[^ ^22^, ^27^, ^28^, ^29^ ^]^ but COP1 knockout mouse model studies suggest that COP1 may also behave as a tumor suppressor via antagonizing proto‐oncogenic activity of c‐Jun and ETV1^[^ ^20^, ^21^, ^30^ ^]^ in some tissues. Therefore, COP1's physiological role in cancer remains controversial and needs further characterization. CSN6 can bind COP1, but the significance of this interaction is largely unknown.

In this study, we characterize the upstream regulators of the FOXO4 in tumorigenesis including EGF, PKB/Akt, CSN6 and COP1. In addition, new FOXO4 downstream targets involved in serine‐glycine‐one‐carbon (SGOC) amino acid metabolism are characterized. Our studies provide important insight into the signaling role of the EGF‐PKB/Akt‐CSN6‐COP1 axis in enhancing ubiquitin‐mediated FOXO4 degradation during tumorigenesis and elucidate a new circuit of regulating FOXO4 transcriptional activity. Our understanding of the role of FOXO4 in inhibiting serine/glycine metabolism of cancer reveals therapeutic opportunities for cancer treatment.

## 2 Results

### 2.1 EGF Signal and CSN6/COP1 Enhance Ubiquitin‐Mediated Degradation of FOXO4

**Figure 1:**
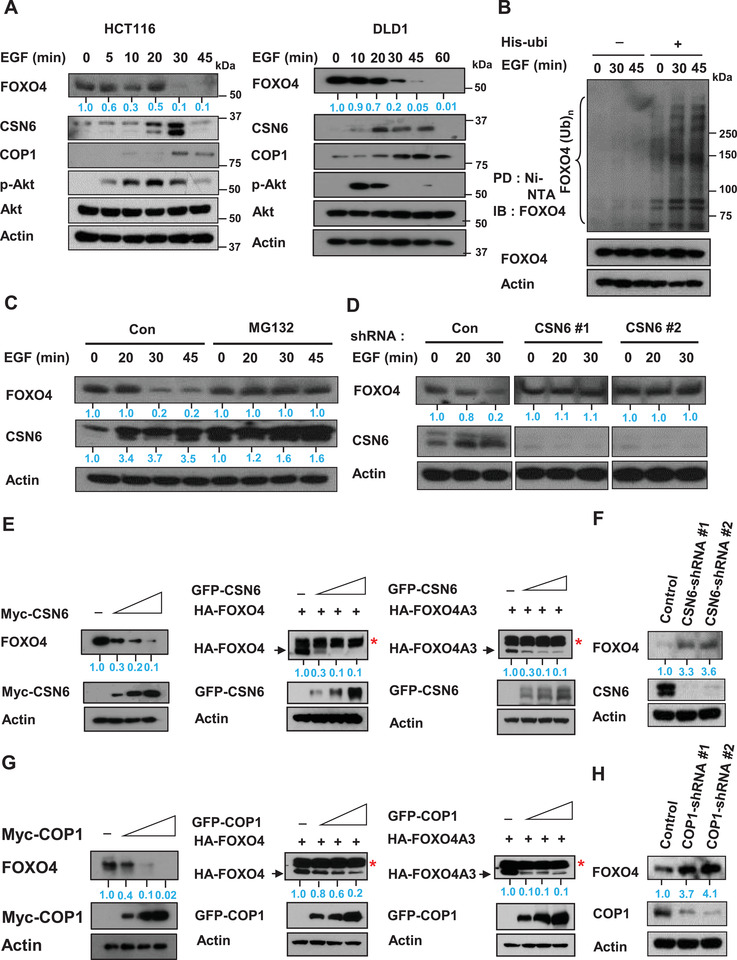
EGF signaling causes FOXO4 down regulation with concurrent elevation of CSN6. A) HCT116 (left) and DLD1 (right) cells were treated with 100 ng mL^−1^ EGF for the indicated minutes. Cell lysates were analyzed by immunoblotting with indicated antibodies. B) HCT116 cells were transfected with His‐ubi plasmid and treated with 100 ng mL^−1^ EGF for the indicated times. Cells were treated with proteasome inhibitor MG132 before collecting lysate. Cells were lysed in denaturing buffer (6 m guanidine‐HCl). The cell lysates were then pull down (PD) with nickel beads and immunoblotted with indicated antibodies. C) HCT116 cells were treated with 100 ng mL^−1^ EGF for the indicated minutes with or without MG132. Lysates were immunoblotted with the indicated antibodies. D) HCT116 cells infected with either CSN6‐shRNA or control shRNA were treated with 100 ng mL^−1^ EGF for the indicated minutes. Cell lysates were immunoblotted with the indicated antibodies. E) DLD1 (left) or 293T cells were cotransfected with the indicated CSN6, FOXO4 (middle), or FOXO4A3 (right) expression vectors. Cell lysates were immunoblotted with indicated antibodies. *Nonspecific. F) Cell lysates of HCT116 cells infected with CSN6 shRNA were immunoblotted with indicated antibodies. G) DLD1 (left) or 293T (middle and right) cells were cotransfected with the indicated plasmids. Cell lysates were immunoblotted with indicated antibodies. *Nonspecific. H) Cell lysates of HCT116 cells infected with COP1 shRNA were immunoblotted with indicated antibodies.

FOXO4 has a tumor suppressive role, but its further upstream regulators remain not well characterized. EGFR signaling is highly activated in cancer;therefore, we sought to determine the dynamics of EGFR activation and FOXO4 regulation. Immunoblotting analysis showed that EGF treatment decreased the steady‐state expression of FOXO4 within 45 min (**Figure** **1**A, Figure S1A, Supporting Information) and accelerated turnover rate of FOXO4 (Figure S1B, Supporting Information) in several CRC cell lines, whereas the EGF induced the expression of CSN6 and COP1, an E3 ligase, within that period of time (Figure 1A, Figure S1A, Supporting Information). As expected, PKB/Akt is activated in response to EGF (Figure 1A, Figure S1A, Supporting Information), suggesting that EGF‐regulated FOXO4 steady‐state expression may involve CSN6, COP1 and PKB/Akt activation. Consistently, the polyubiquitination level of FOXO4 is increased in response to EGF treatment, suggesting that EGF‐regulated FOXO4 down regulation involves a polyubiquitination process (Figure 1B). Further, EGF‐mediated down regulation of FOXO4 is in a time‐dependent manner and can be antagonized by proteasome inhibitor MG132 (Figure 1C).

Importantly, EGF‐mediated down regulation of FOXO4 can be reversed by the knockdown of CSN6 (Figure 1D), suggesting the involvement of CSN6 during this process. We then found that CSN6 decreased the steady‐state expression of FOXO4 in a dose‐dependent manner in several CRC cell lines (Figure 1E, Figure S2A, Supporting Information). Interestingly, CSN6 can also reduce the steady‐state expression of FOXO4A3 (Figure 1E), which has all the PKB/Akt phosphorylation sites mutated. In line with this finding, Western blotting showed that knockdown of CSN6 by shRNA increased the steady‐state expression of FOXO4 (Figure 1F). To address if COP1, which is linked to CSN6 regulation, has any role, Western blotting revealed that COP1 also reduced the steady‐state expression of FOXO4 and FOXO4A3 in a dose‐dependent manner (Figure 1G, Figure S2A, Supporting Information). Knockdown of COP1 by shRNA increased the steady‐state expression of FOXO4 (Figure 1H). Importantly, COP1 knockdown has compromised EGF‐meditated turnover rate of FOXO4 (Figure S2B, Supporting Information). Taken together, these results indicate that EGF signaling elevated CSN6 and COP1 to cause down regulation of FOXO4 regardless of its PKB/Akt‐mediated phosphorylation status.

### 2.2 CSN6 Enhances Ubiquitin‐Mediated Degradation of FOXO4 through K48 Link

**Figure 2:**
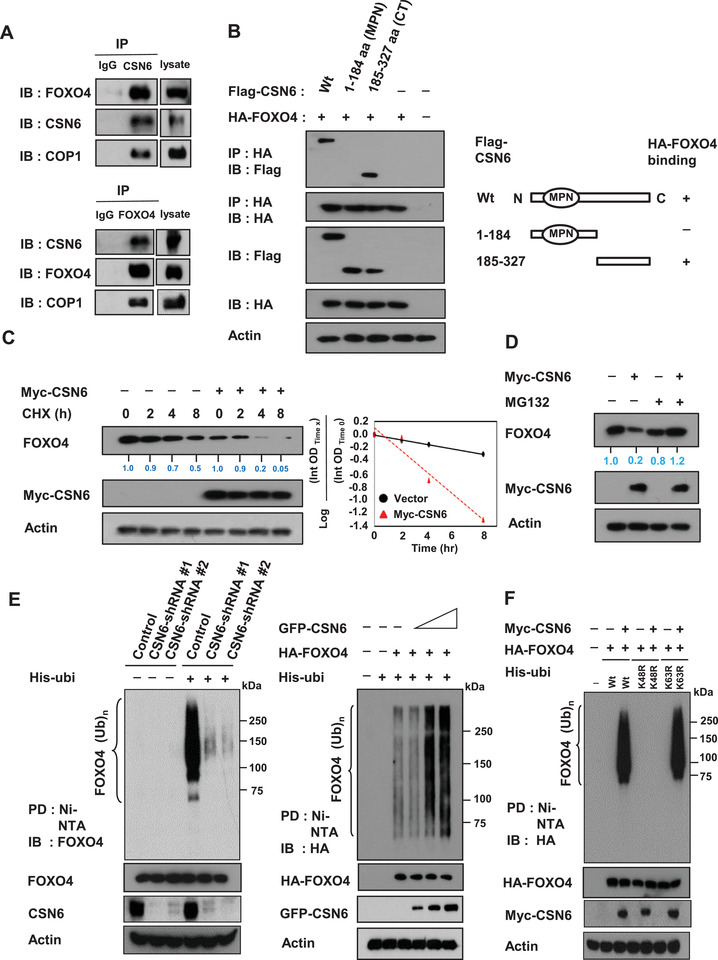
CSN6 increases FOXO4 turnover rate and enhances FOXO4 ubiquitination. A) HCT116 cell lysates were immunoprecipitated with either control rabbit IgG, CSN6, or FOXO4 antibodies followed by immunoblotting with indicated antibodies. B) CSN6 full length wild‐type, N‐terminal (aa 1‐184) or C‐terminal (aa 185‐327) was transfected into 293T cells. Cell lysates were immunoprecipitated with anti‐HA and immunoblotted with anti‐Flag for binding studies. C) SW480 cells transfected with the indicated plasmids were treated with cycloheximide (CHX) (100 µg mL^−1^) for the indicated hours. Cell lysates were immunoblotted with indicated antibodies. The turnover rate of FOXO4 is shown. D) SW480 cells transfected with indicated plasmids were treated with or without proteasome inhibitor MG132. Lysates were immunoblotted with indicated antibodies. E) Cells transfected with the indicated CSN6‐shRNA (left, HCT116 cells) or CSN6 plasmids (right, 293T cells) were treated with 5 µg mL^−1^ MG132 (Sigma) for 6 h before harvesting. Cells were lysed in guanidine‐HCl containing buffer and cell lysates were then pull down (PD) with nickel beads and immunoblotted with indicated antibodies. F) The 293T cells were cotransfected with indicated plasmids. Cells were treated with 5 µg mL^−1^ MG132 for 6 h before harvesting. Cells were lysed in guanidine‐HCl containing buffer. The cell lysates were then pull down (PD) with nickel beads and immunoblotted with anti‐HA.

On the basis of the above findings, we hypothesized that CSN6, COP1, and FOXO4 have an interactive or regulatory relationship. We found that CSN6, COP1, and FOXO4 form complexes dynamically under EGF stimulation as evidenced by coelution from a gel filtration experiment (Figure S3A, Supporting Information). Next, CSN6 immunoprecipitation experiments showed endogenous interaction of the three proteins in cells (**Figure** **2**A). We also documented that CSN6 and COP1 interact with FOXO4 directly based on a GST‐FOXO4 pull down assay and in situ proximity‐ligation assay (Figure S3B,C and Figure S4, Supporting Information).

Next, we mapped the structural domains of CSN6 required for its interaction with FOXO4. Results showed that FOXO4 bound to the C‐terminus of CSN6 (aa 185‐327 containing) but not to the N‐terminus (aa 1‐184, containing the MPN domain; Figure 2B).

We further investigate how CSN6 downregulates FOXO4. A qRT‐PCR assay indicated that CSN6 regulated FOXO4 post‐transcriptionally as CSN6 expression led to FOXO4 protein reduction but had no impacts on mRNA expression of FOXO4 (Figure S5A,B, Supporting Information). CSN6 overexpression increased the turnover rate of FOXO4 (Figure 2C, Figure S5C, Supporting Information). Further, CSN6‐mediated FOXO4 down regulation was rescued by the proteasome inhibitor MG132 (Figure 2D, Figure S5D, Supporting Information). We then found that CSN6 knockdown reduced the ubiquitination level of FOXO4 (Figure 2E), whereas overexpression of CSN6 increased the ubiquitination level of FOXO4 in a dose‐dependent manner (Figure 2E). We showed that CSN6 employed wild‐type (wt) His‐Ubi or His‐Ubi K63R mutant to cause ubiquitination of FOXO4 but was unable to use His‐Ubi K48R mutant to facilitate FOXO4 ubiquitination (Figure 2F), demonstrating that CSN6‐mediated ubiquitination of FOXO4 is a K48 linkage (Figure 2F), which generally leads to protein degradation.^[^ ^31^ ^]^ Our data indicates that CSN6 enhances ubiquitin‐mediated degradation of FOXO4 through K48 link ubiquitination, thereby downregulating FOXO4.

### 2.3 COP1 Is Involved in CSN6‐Mediated Ubiquitination of FOXO4

**Figure 3:**
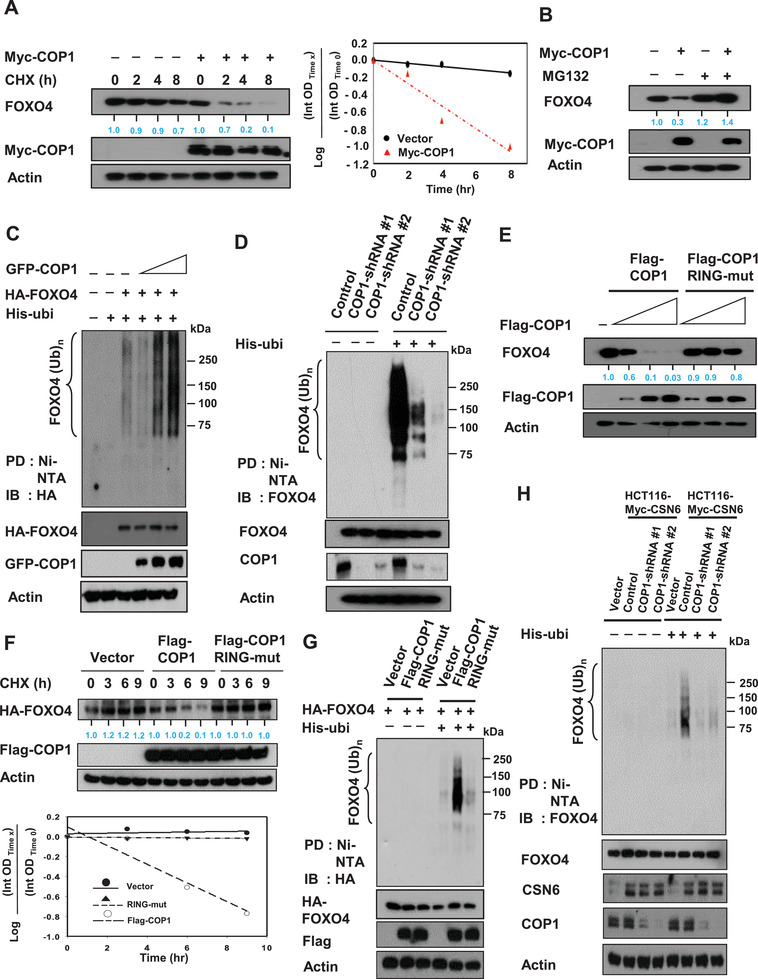
COP1 is involved in CSN6‐mediated ubiquitination of FOXO4. A) SW480 cells transfected with the indicated expression vectors were treated with cycloheximide (CHX) (100 µg mL^−1^). Cell lysates were immunoblotted with indicated antibodies. The turnover rate of FOXO4 is shown. B) SW480 cells transfected with either Myc‐COP1 or vector control was treated with proteasome inhibitor MG132. Lysates were immunoblotted with indicated antibodies. C,D) 293T cells transfected with C) the indicated plasmids or HCT116 cells infected with D) COP1 shRNA were treated with 5 µg mL^−1^ MG132 (Sigma) for 6 h before harvesting. Cells were lysed in guanidine–HCl containing buffer. The cell lysates were then pull down (PD) with nickel beads Ni‐NTA and immunoblotted with the indicated antibodies. E) DLD1 cells were transfected with the indicated expression vectors. Lysates were immunoblotted with indicated antibodies. F) 293T cells were transfected with the indicated expression vectors. The cells were treated with cycloheximide (CHX) (100 µg mL^−1^) for the indicated hours. Cell lysates were immunoblotted with indicated antibodies. The turnover rate of FOXO4 is shown. G) 293T cells were transfected with the indicated expression vectors. Cells were treated with MG132 for 6 h before harvesting, and lysed in denaturing buffer (6 m guanidine‐HCl). The cell lysates were then pull down (PD) with nickel beads and immunoblotted with anti‐HA. H) Myc‐CSN6 overexpressing HCT116 cells infected with either COP1 shRNA were treated with MG132 for 6 h before harvesting. HCT116 vector cells were used as a control. Cells were lysed in denaturing buffer (6 m guanidine‐HCl). The cell lysates were then pull down (PD) with nickel beads and immunoblotted with indicated antibodies.

CSN6 targets proteins for ubiquitin‐mediated degradation. Regulation of E3 ligase activity is important for the activity of CSN6. To further investigate how CSN6 regulates FOXO4 ubiquitination, we examined whether the E3 ligase COP1, which is known to associate with CSN6, has a role in this process. Compared with control cells, cells transfected with COP1 had an accelerated FOXO4 turnover rate (**Figure** **3**A, Figure S6A, Supporting Information); qRT‐PCR data indicates that COP1 status did not affect FOXO4 mRNA levels (Figure S6B, Supporting Information). But COP1 is involved in the steady state expression of FOXO4 (Figure S6C, Supporting Information). COP1‐mediated FOXO4 down regulation can be rescued by the proteasome inhibitor MG132 (Figure 3B, Figure S6D, Supporting Information). Overexpression of COP1 increased the ubiquitination level of FOXO4 in a dose‐dependent manner (Figure 3C), whereas COP1 knockdown by shRNA reduced the ubiquitination level of FOXO4 (Figure 3D). We then showed that the RING domain of COP1 is critical for regulating FOXO4 degradation because the COP1 RING mutant (C136S/C139S) was not able to downregulate steady‐state expression of FOXO4 in a dose‐dependent manner (Figure 3E, Figure S6E, Supporting Information). Congruently, COP1 RING mutant (C136S/C139S) failed to increase the turnover of FOXO4 (Figure 3F) and was unable to accelerate subsequent FOXO4 ubiquitination (Figure 3G). These data indicate that COP1 regulates FOXO4 ubiquitination via its RING domain. Further, to demonstrate that CSN6 and COP1 collaborate to regulate FOXO4 ubiquitination, we performed a CSN6‐mediated FOXO4 ubiquitination experiment under COP1 knockdown condition and found that CSN6‐mediated FOXO4 ubiquitination was diminished in the presence of COP1 shRNA (Figure 3H), suggesting the requirement of COP1 during the process. Together, these data indicated that CSN6‐COP1 axis is involved in promoting FOXO4 ubiquitination.

### 2.4 CSN6/COP1‐Mediated FOXO4 Ubiquitination Requires the Physical Interaction between COP1 and VP Motif of FOXO4

**Figure 4:**
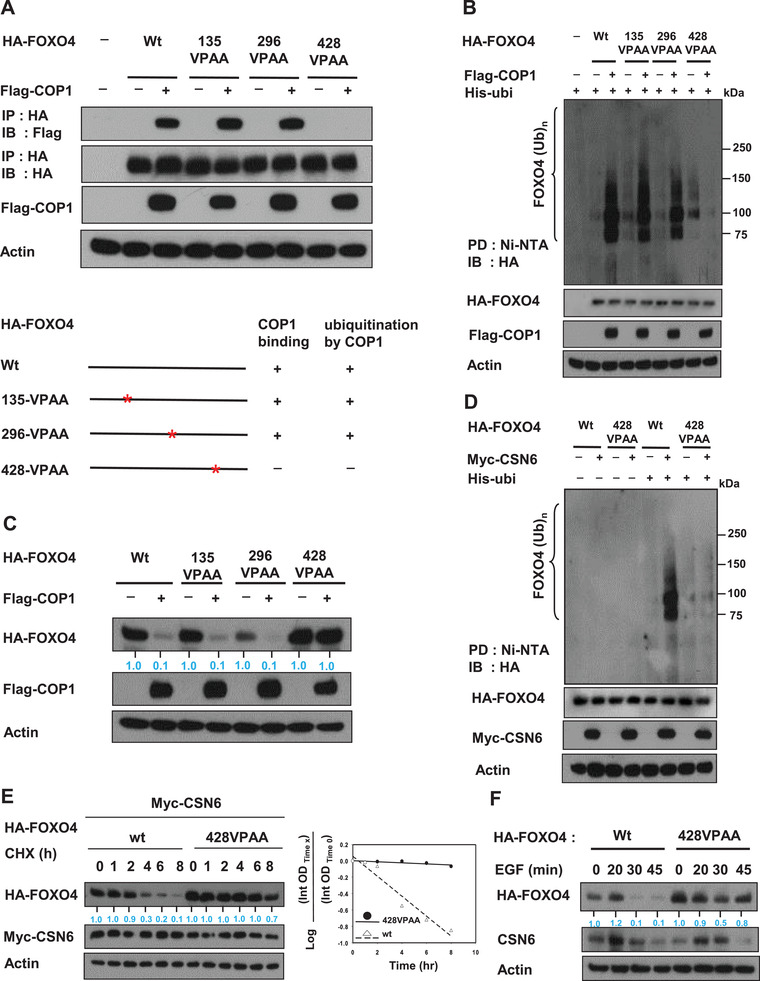
CSN6/COP1‐mediated FOXO4 ubiquitination requires physical interaction between COP1 and VP motif of FOXO4. A) The 293T cells were transfected with indicated HA‐FOXO4 VPAA plasmids. Cell lysates were immunoprecipitated with anti‐HA and immunoblotted with indicated antibodies. COP1's impacts on these FOXO4 VPAA plasmids are demonstrated. B) The 293T cells were cotransfected with indicated plasmids. Cells were treated with 5 µg mL^−1^ MG132 (Sigma) for 6 h before harvesting. Cells were lysed in guanidine‐HCl containing buffer. The cell lysates were then pull down (PD) with nickel beads and immunoblotted with anti‐HA. C) 293T cells were co‐transfected with indicated plasmids. Cell lysates were immunoblotted with indicated antibodies. D) The 293T cells were cotransfected with indicated plasmids. Cells were treated with 5 µg mL^−1^ MG132 (Sigma) for 6 h before harvesting. Cells were lysed in guanidine‐HCl containing buffer. The cell lysates were then pull down (PD) with nickel beads and immunoblotted with anti‐HA. E) Myc‐CSN6 overexpressing HCT116 cells were transfected with indicated plasmids. Cells were treated with cycloheximide (CHX) (100 µg mL^−1^) for the indicated time. Cell lysates were immunoblotted with indicated antibodies. The turnover rate is shown. F) HCT116 cells were transfected with either HA‐FOXO4 or HA‐FOXO4 428VPAA mutant. Cells were treated with 100 ng mL^−1^ EGF for the indicated minutes. Cell lysates were immunoblotted with indicated antibodies.

COP1 binds to target proteins containing the VP motifs.^[^ ^32^ ^]^ We analyzed the FOXO4 peptide sequence and found that several VP motifs (135VP, 296VP, 428VP) are present in FOXO4 (Figure S7A,B, Supporting Information). We predicted that abolishing these potential binding sites by mutating the VP motif to alanine (VP→AA) would help identify the correct binding sites. Coimmunoprecipitation studies indicated that the FOXO4 (428VP→AA) mutant but not other indicated mutants (135VP→AA, 296VP→AA) lost its binding affinity for COP1 (**Figure** **4**A), suggesting that the 428VP sequence of FOXO4 is the binding site for COP1. In line with the binding requirement for COP1‐mediated FOXO4 degradation, the FOXO4 (428VP→AA) mutant was resistant to COP1‐mediated ubiquitination (Figure 4B). Importantly, COP1 can reduce the steady‐state expression of FOXO4 (135VPAA) and FOXO4 (296VPAA) but not FOXO4 (428VPAA) (Figure 4C), suggesting that binding at 428VP sequence is critical for the COP1‐mediated FOXO4 degradation. To further confirm the CSN6‐COP1 axis is involved in regulating FOXO4 ubiquitination, we investigate the ubiquitination level of FOXO4 (428VP→AA) mutant in the presence of CSN6. As expected, wt FOXO4 ubiquitination was enhanced by CSN6. However, the FOXO4 (428VP→AA) mutant was resistant to CSN6's activity in terms of ubiquitination (Figure 4D). Consistently, the turnover of wt FOXO4 was accelerated by CSN6, while the turnover of FOXO4 (428VP→AA) mutant was not affected by CSN6 (Figure 4E). To put this observation in the EGF signaling context, FOXO4 (428VP→AA) mutant was not downregulated by EGF when compared with wt FOXO4 (Figure 4F). Together, these results demonstrated that CSN6‐mediated down regulation of FOXO4 requires the protein–protein interaction between COP1 and FOXO4 428VP motif.

### 2.5 Deregulation of CSN6‐FOXO4 Axis Rewires Metabolic Programming via Enhancing Glucose Uptake and Promotes the Expression of SGOC Genes

The transcriptional activity role of FOXO4 is documented in its activity in regulating genes involved in oxidative stress, cell proliferation, apoptosis, and metabolism. We have shown that CSN6 decreases FOXO4 stability. To address the biological significance behind this regulation, we examined the transcriptional activity of FOXO4 through reporter gene assay. CSN6‐mediated FOXO4 destabilization translated into decreased FOXO4 transcriptional activity, as evidenced by reducing FOXO4 luciferase reporter gene activity (Figure S8A, Supporting Information). COP1 is also reducing FOXO4 luciferase reporter gene activity (Figure S8B, Supporting Information). As FOXOs are involved in regulating cancer metabolism, a hallmark of cancer, we hypothesized that CSN6‐FOXO axis may have impact on cancer metabolism. Metabolomics studies indicate that several metabolites, including creatine, glutathione disulfide, and SAM are deregulated under CSN6 knockdown (Figure S9A,B, Supporting Information). LDHA activity is downregulated under CSN6 knockdown condition (Figure S9C, Supporting Information). Data mining from TCGA also showed that CSN6 expression level is positively correlated with the expression of SGOC genes involved in serine‐glycine metabolism, including PHGDH, PSPH, SHMT1, and PSAT1 (Figure S10, Supporting Information).

**Figure 5:**
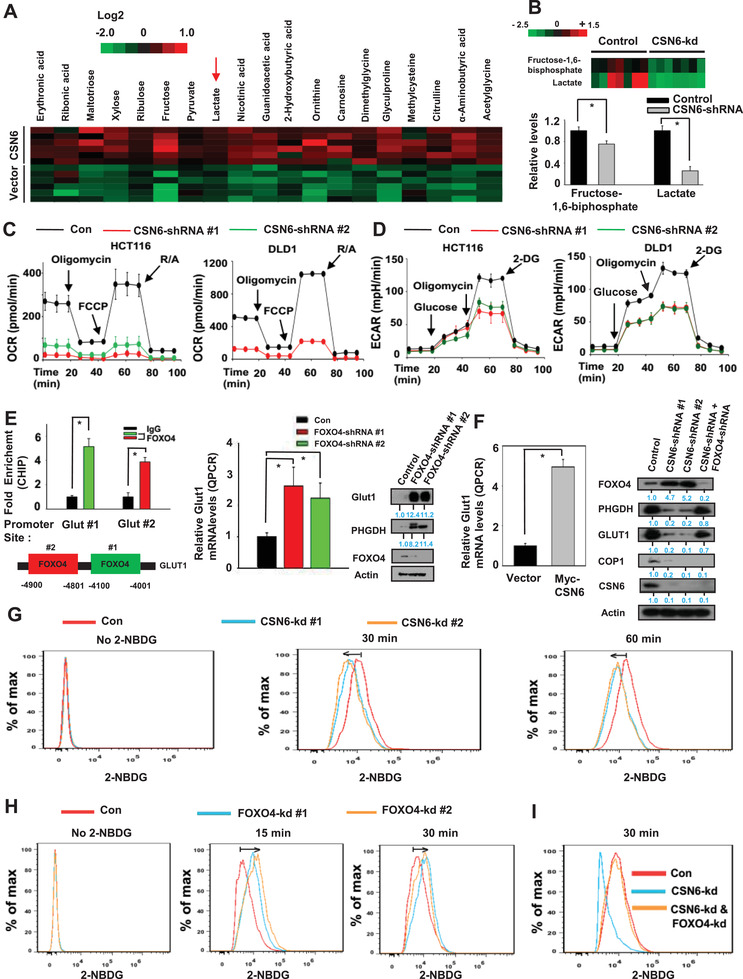
CSN6–FOXO4 axis regulates glucose uptake. A) Changes of metabolites were determined by mass spectrometry in HCT116 vector and CSN6 overexpressing cells. B) Knockdown of CSN6 reduces fructose‐1,6‐biphosphate and lactate production. Metabolic analysis determined by mass spectrometry in HCT116 cells infected with control shRNA or CSN6 shRNA. Bars represent average ± s.d., *n* = 7, student's t‐test, *P < 0.05. C,D) Oxygen consumption rates (OCRs) and extracellular acidification rates (ECARs) were measured in CSN6 knockdown HCT116 cells. Values are average ± s.d., *n* = 3. E) ChIP‐PCR analysis of Glut1 promoter in HCT116 cells using anti‐FOXO4 antibody and PCR primers. Enrichment of FOXO4 binding on the Glut1 gene promoter was presented as a bar graph (left, top). IgG was used as a control. Two putative FOXO4‐binding sites in Glut1 promoter are indicated (left panel, bottom). RT‐qPCR analysis of Glut1 in FOXO4 shRNA infected HCT116 cells (middle panel). Lysates of HCT116 cells infected with FOXO4 shRNA were immunoblotted with indicated antibodies (right panel). Bars represent average ± s.d., *n* = 3, student's t‐test (left panel) and one‐way ANOVA (right panel), *P < 0.05. F) Real‐time qPCR analysis of Glut1 in Myc‐CSN6 expressing HCT116 cells (left panel). Lysates of HCT116 cells infected with indicated shRNA were immunoblotted with indicated antibodies (right panel). Bars represent average ± s.d., *n* = 3, student's t‐test, *P < 0.05. G,H) HCT116 control and HCT116 CSN6 or FOXO4 knockdown cells were incubated with 2‐NBDG for the indicated period of time. 2‐NBDG uptake was measured by flow cytometry. I) Indicated knockdown cells were incubated with 2‐NBDG for 30 min. 2‐NBDG uptake was determined by flow cytometry.

Metabolomic analysis by mass spectrometry showed that CSN6 overexpression causes elevation of several metabolites including lactate (**Figure** **5**A, Figures S11 and S12, Supporting Information). Another metabolomic analysis by mass spectrometry demonstrated that CSN6 knockdown leads to reduction of lactate and fructose‐1,6‐bisphosphate, and the significance of the changes in metabolite levels was documented (Figure 5B, Figures S13 and S14, Supporting Information). Further, we performed seahorse analysis and showed that knockdown of CSN6 decreased mitochondrial respiration as indicated by oxygen consumption rate (OCR) (Figure 5C) and extracellular acidification rate (ECAR) (Figure 5D), an indicator of glycolysis, in two colorectal cancer cell lines.

CHIP assays showed that FOXO4 is binding to Glut1 (glucose transporter 1) promoter (Figure 5E) to affect gene expression of Glut1 negatively as demonstrated by the elevated gene expression and protein level of Glut1 under the condition of FOXO4 knockdown (Figure 5E, Figure S15A, Supporting Information). In the same protein assay, it seems that phosphoglycerate dehydrogenase (PHGDH) involved in serine‐glycine‐one‐carbon (SGOC) amino acid metabolism was elevated also when FOXO4 is knocked down by shRNA (Figure 5E). As CSN6 mitigates the expression level of FOXO4, we showed that CSN6 overexpression leads to increased gene expression of Glut1 (Figure 5F, Figure S15A, Supporting Information). In contrast, protein analysis demonstrated that CSN6 knockdown reduced the expression of Glut1 while increased the expression of FOXO4 (Figure 5F). This impact of CSN6 knockdown on Glut1 expression was reversed when FOXO4 was knockdown at the same time (Figure 5F). In the same protein blot, the expression of COP1 and PHGDH expression was affected accordingly (Figure 5F), consistent with CSN6's involvement in the expression of SGOC genes (Figure S10, Supporting Information).

As CSN6‐FOXO4 axis impacts on the expression of Glut1, biochemical assays that quantitates the glucose uptake (consumption) by assessing uptake of (2‐(*N*‐(7‐nitrobenz‐2‐oxa‐1,3‐diazol‐4‐yl)amino)‐2‐deoxyglucose (2‐NBDG)), a green fluorescent glucose analog, additionally demonstrated that CSN6 knockdown inhibited 2‐NBDG uptake (Figure 5G), while FOXO4 knockdown increased 2‐NBDG uptake (Figure 5H). Again, the impact of CSN6 knockdown on 2‐NBDG uptake was reversed when FOXO4 was knockdown at the same time (Figure 5I). These data suggest again that CSN6‐COP1‐FOXO4 axis is involved in regulating glucose transporting, thereby affecting glucose uptake/consumption.

**Figure 6:**
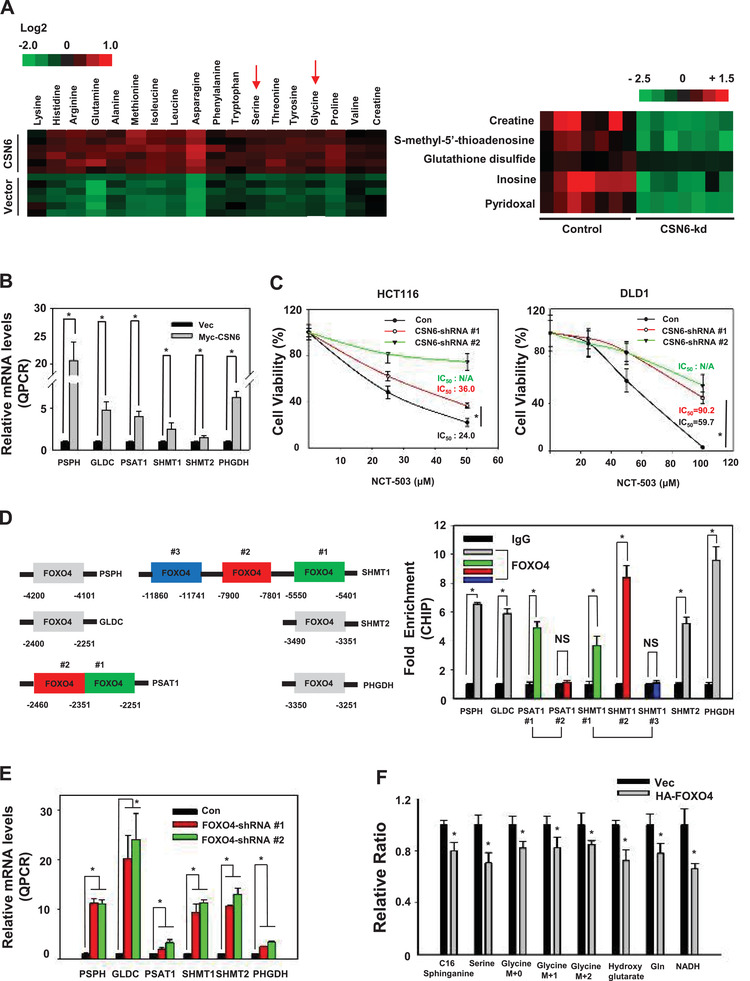
CSN6‐FOXO4 axis regulates the expression of serine–glycine one carbon genes. A) Changes of serine and glycine metabolites were determined by mass spectrometry in HCT116 vector and CSN6 overexpressing cells (left panel). Changes of SGOC pathway‐related metabolites, including glutathione disulfides and pyridoxal, determined by mass spectrometry in HCT116 cells infected with control shRNA or CSN6 shRNA (right panel). B) Real‐time quantitative PCR analysis of serine pathway genes in Myc‐CSN6‐expressing HCT116 cells. Bars represent average ± s.d., *n* = 3, two‐tailed Student's t‐test, *P < 0.05. C) Indicated cell viability was measured by CCK8 at the indicated concentrations of NCT‐503. Values represent average ± s.d., *n* = 8, two‐tailed t‐test, *P < 0.05. D) ChIP‐PCR analysis of HCT116 cells using anti‐FOXO4 antibody and PCR primers. Promoter of the SGOC pathway genes contains putative FOXO4‐binding sites (left panel). Enrichment of FOXO4 binding on the serine pathway gene promoter was presented as a bar graph (right panel). IgG was used as a control. Bars represent average ± s.d., *n* = 3, two‐tailed Student's t‐test, *P < 0.05. E) Real‐time quantitative PCR analysis of serine pathway genes in FOXO4 knockdown HCT116 cells. Bars represent average ± s.d., *n* = 3, two‐tailed Student's t‐test, *P < 0.05. F) Incorporation of carbon‐13 (^13^C) from [U‐^13^C] glucose (11 × 10^−3^ m) into the indicated metabolites at 24 h in HCT116 cells. The data are presented as the means ± s.d. Bars represent average ± s.d., *n* = 3, two‐tailed Student's t‐test, *P < 0.05.

Metabolomic analysis by mass spectrometry also demonstrated that CSN6 overexpression leads to elevation of several metabolites such as serine and glycine (**Figure** **6**A, Figures S11 and S12, Supporting Information). Another metabolomic analysis by mass spectrometry demonstrates that CSN6 knockdown reveals reduction of creatine and pyridoxal (Figure 6A, Figures S13 and S14, Supporting Information). We then sought to analyze the expression of genes involved in SGOC pathway. We showed that CSN6 expression elevated the gene expression of SGOC pathway genes as assayed by real‐time quantitative PCR (Figure 6B). We then performed cell viability assays and found that NCT‐503, a new small‐molecule PHGDH inhibitor, inhibited the growth of control cell with a lower NCT‐503 IC50, while CSN6 knockdown cells were less responsive and showed higher NCT‐503 IC50 (Figure 6C).

Promoter search identified several FOXO4 binding sites located at the promoters of PHGDH, PSAT1, PSPH, SHMT1, GLDC and SHMT2 (Figure 6D). Quantitation of the binding between FOXO4 and SGOC gene promoters through CHIP PCR assay revealed that the binding of FOXO4 to SGOC gene promoters was enriched when compared with that of immunoglobulin G (IgG) (Figure 6D). Importantly, FOXO4 knockdown leads to elevation of gene expression of SGOC pathway genes as demonstrated by real‐time quantitative PCR analysis, suggesting that FOXO4 binds to SGOC gene promoters to suppress gene expression (Figure 6E, Figure S15B, Supporting Information). Metabolite tracing revealed that FOXO4 expression leads to reduced production of SGOC metabolites including serine and glycine (Figure 6F). These data imply that CSN6 possibly regulates the gene expression of SGOC genes and impacts SGOC amino acid metabolism through its negative impact on FOXO4 that transcriptionally suppresses the gene expression of SGOC genes.

### 2.6 PKB/Akt‐Mediated CSN6 Phosphorylation Enhances COP1‐Regulated FOXO4 Ubiquitination and Subsequent Elevation of SGOC Genes

**Figure 7:**
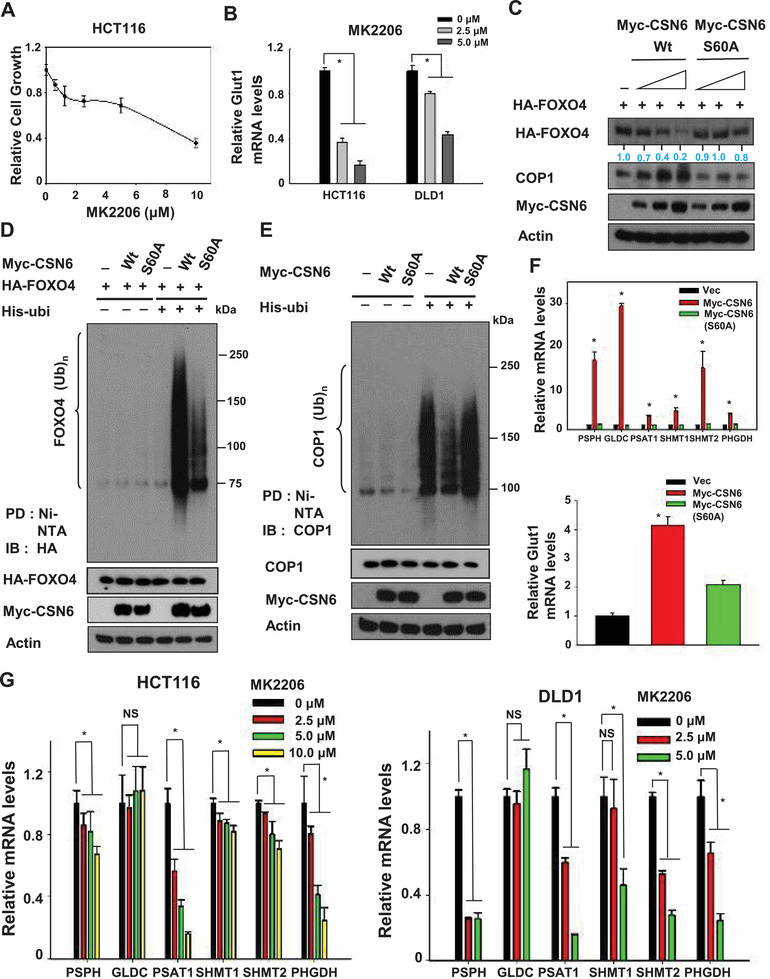
PKB/Akt signal activates CSN6 via phosphorylation to regulate FOXO4‐mediated gene expression of SGOC network. A) HCT116 cells were treated with different concentrations of MK2206 and cell viability was measured by CCK8. Values represent average ± s.d., *n* = 8. B) Real‐time quantitative PCR analysis of Glut1 in indicated cell lines was performed after the treatment of MK2206. Bars represent average ± s.d., *n* = 3, one‐way ANOVA, *P < 0.05. C) 293T cells were transfected with the indicated CSN6 and CSN6 S60A expression vectors. Cell lysates were immunoblotted with indicated antibodies. D,E) 293T cells were transfected with the indicated expression vectors. Cells were treated with MG132 for 6hr before harvesting, and lysed in denaturing buffer (6 m guanidine–HCl). The cell lysates were then pull down (PD) with nickel beads and immunoblotted with indicated antibodies. F) Real‐time quantitative PCR analysis of serine pathway genes (top) and Glut1 gene (bottom) in HCT116 transfected with indicated expressing plasmids. Bars represent average ± s.d., *n* = 3, one‐way ANOVA, *P < 0.05. G) Real‐time quantitative PCR analysis was performed to measure mRNA levels of SGOC pathway genes in HCT116 (left) and DLD1 (right) cells treated with MK2206. Bars represent average ± s.d., *n* = 3, one‐way ANOVA, *P < 0.05.

Since EGF decreases the stability of FOXO4, we reasoned that the EGF axis would have a role in FOXO4 regulation. PKB/Akt is an important mediator of EGF signaling. We found that PKB/Akt inhibitor MK2206 inhibits cell growth (**Figure** **7**A) and reduces the gene expression of Glut1 (Figure 7B), a target of FOXO4. S60 of CSN6 is phosphorylated by PKB/Akt^[^ ^16^ ^]^ and is critical for CSN6 stabilization. To determine whether the PKB/Akt‐mediated CSN6 phosphorylation affects FOXO4 stability, we examined the steady‐state expression of FOXO4 and COP1 in the presence of CSN6S60A, a construct with no PKB/Akt phosphorylation site. Interestingly, wt CSN6 increased the turnover rate of FOXO4 to destabilize FOXO4 in a dose‐dependent manner, while CSN6 S60A had lost such a capability (Figure 7C). Also, the ubiquitination level of FOXO4 is less responsive to the CSN6S60A mutant's impact when compared with wt CSN6 (Figure 7D). To further demonstrate the CSN6‐COP1‐FOXO4 circuit, we then showed that wt CSN6 reduced the ubiquitination levels of COP1, but CSN6S60A mutant has lost such a characteristic (Figure 7E). Congruently, CSN6S60A mutant was unable to promote the gene expression of suppressed targets of FOXO4, including SGOC pathway genes and Glut1 (Figure 7F).

As the PKB/Akt‐CSN6‐FOXO4 axis in regulating SGOC gene expression has suggested, PKB/Akt inhibitor MK2206 was able to inhibit the gene expression of SGOC genes in a dose‐dependent manner (Figure 7G). Since MDM2 was shown as an E3 ligase for FOXO4 proteins,^[^ ^33^, ^34^ ^]^ we examined whether MDM2 is involved in CSN6‐mediated FOXO4 degradation. Our data showed that CSN6‐mediated FOXO4 degradation was not affected by MDM2 knockdown, suggesting a MDM2‐independent process (Figure S16, Supporting Information). These results indicate that PKB/Akt‐mediated CSN6 phosphorylation is critical in stabilizing COP1, thereby enhancing ubiquitination level of FOXO4. Thus, EGF‐PKB/Akt‐CSN6‐COP1 axis is involved in FOXO4 ubiquitination and subsequent activation of SGOC genes.

### 2.7 CSN6‐FOXO4 Axis Is Critical for Tumorigenicity in Xenograft Mouse Model

We further confirmed that CSN6‐FOXO4 link could affect cell proliferation. CSN6 knockdown hindered cell growth, soft agar colony formation, and tumorigenicity (Figure S17A–D, Supporting Information) compared with control cells. On the other hand, the FOXO4 knockdown cells have a faster rate of cell proliferation and soft agar colony formation (Figure S17A,B, Supporting Information). CSN6 knockdown‐mediated cell growth inhibition, soft agar colony formation reduction can be rescued by FOXO4 knockdown (Figure S17A,B, Supporting Information).

To demonstrate the impact of CSN6‐COP1 axis in regulating FOXO4 and subsequent gene expression of SGOC genes in vivo, we performed mouse xenograft cancer studies and demonstrated that CSN6 knockdown suppressed tumor growth (Figure S17C, Supporting Information). Importantly, in these mouse xenograft cancer model studies, control tumors contain low levels of FOXO4 and COP1 while demonstrate relatively high levels of PHGDH and Glut1 (Figure S17D, Supporting Information). As expected, the expression of PHGDH, Glut1, and COP1 was diminished in CSN6 knockdown xenograft tumors with concurrent elevation of FOXO4 (Figure S17D, Supporting Information). Significantly, as the CSN6–FOXO4 axis is crucial in regulating gene expression of Glut1 and SGOC genes, these genes were reduced in CSN6 knockdown tumors (Figure S17E, Supporting Information). Together, the regulatory circuit of CSN6‐FOXO4 axis could be recapitulated in mouse xenograft cancer model, and level of FOXO4 deregulation plays roles in affecting the outcome of tumorigenicity.

### 2.8 Deregulation of CSN6‐COP1‐FOXO4 Axis Is Correlated with Poor Survival in Human Colorectal Cancer

**Figure 8:**
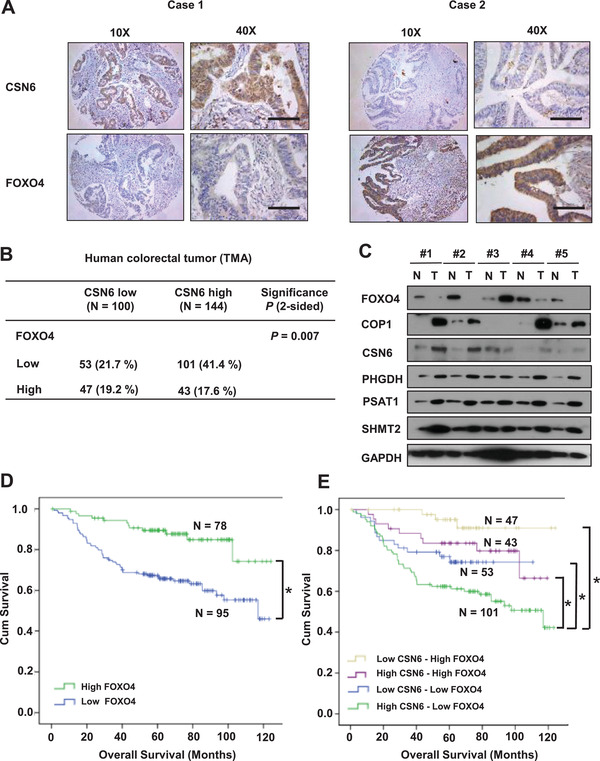
Validation of CSN6–FOXO4 deregulation in human colorectal cancer samples. A) Representative IHC staining for CSN6 and FOXO4 in human TMAs. Case 1 is representative of a patient with CSN6‐high colon cancer. Case 2 is representative of a patient with CSN6‐low colon cancer. Scale bar = 100 µm B) Quantification of staining intensities of indicated protein from sections in A). CSN6 and FOXO4 show a negative correlation. C) Adjacent (N) and tumor (T) tissues of colorectal cancer patients were collected and lysates were analyzed by immunoblotting with indicated antibodies. D,E) Kaplan‐Meier curves of relapse‐free survival time based on CSN6 and FOXO4 expression from TMA analysis. Log‐rank test, *P < 0.001.

To examine and confirm the relationship among CSN6, COP1, and FOXO4 in human cancers, we performed IHC staining on a tissue microarray (TMA) from our human colon cancer cohort (Table S1, Supporting Information) to assess the expression of these proteins (**Figure** **8**A). CSN6 and FOXO4 showed a significant reverse correlation in IHC staining intensity (Figure 8A), while CSN6 showed a significant positive correlation with PSAT1 and SHMT2 in these CRC tissues (Figure S18, Supporting Information). These tumor samples were classified into four groups on the basis of the expression levels: high CSN6 and low FOXO4 expression, high CSN6 and high FOXO4 expression, low CSN6 and low FOXO4 expression, and low CSN6 and high FOXO4 (Figure 8B). The statistical analysis is significant regarding the reverse relationship between CSN6 and FOXO4. In addition, CSN6, COP1, FOXO4, PHGDH, PSAT1, and SHMT2 demonstrated a significant correlation in staining intensity in five pairs of primary tumor/normal tissue samples as expected (Figure 8C), i.e., high CSN6 expressing tumors have lower levels of FOXO4 with concurrent high expression of COP1, PHGDH, PSAT1, and SHMT2.

Significantly, we demonstrated that low level of FOXO4 is correlated with poor survival of CRC patients based on the Kaplan–Meier analysis (Figure 8D). Furthermore, on the basis of the expression status of both CSN6 and FOXO4 the Kaplan–Meier analysis results indicated that patients with high CSN6 and low FOXO4 expression tend to exhibit the poorest overall survival when compared with other groups (Figure 8E). In summary, these data indicate that CSN6–COP1‐FOXO4 axis is deregulated in CRC. These data also demonstrate that both FOXO4 and CSN6 can serve as molecular markers of CRC.

## 3 Discussion

Although the FOXO family members, including FOXO4, are involved in many biological activities, our knowledge about upstream regulators and downstream targets of FOXO4 is still very limited. EGF‐PKB/Akt signaling is activated in many types of cancer. CSN6 is overexpressed in many types of cancer and is a critical ubiquitination regulator involved in cell proliferation^[^ ^16^, ^29^ ^]^ and promoting tumorigenesis. Here we show that EGF, PKB/Akt, CSN6, and COP1 have oncogenic activities in regulating FOXO4. COP1 is identified here as an E3 ubiquitin ligase that binds and destabilizes FOXO4. Notably, FOXO4 inhibits SGOC pathway metabolic reprogramming of cancer. This study provides insight into FOXO4 upstream regulatory circuit and elucidates how oncogenical signal in interfering FOXO4's downstream inhibitory activity toward SGOC pathway to promote cancer development.

Our data show that EGF stimulates PKB/Akt and causes FOXO4 down regulation in a time‐dependent manner with concurrent elevation of CSN6 and COP1 (Figure 1A). EGF treatment promotes the ubiquitination levels of FOXO4 (Figure 1B), indicating EGF‐PKB/Akt‐CSN6‐COP1 link in regulating FOXO4 poly‐ubiquitination and steady sate expression. Our observations show that role of PKB/Akt in regulating FOXO4 stability seems to be at the level of FOXO4's upstream regulator—CSN6 rather than PKB/Akt phosphorylation of FOXO4. First, we have shown that S60 of CSN6 can be phosphorylated by PKB/Akt^[^ ^16^ ^]^ and that PKB/Akt enhances the steady‐state expression of CSN6.^[^ ^16^ ^]^ This S60 site is located in CSN6's MPN (Mpr1p and Pad1p N‐terminal) domain, a domain found in the N‐terminus of yeast Mpr1 and Pad1 proteins.^[^ ^17^, ^35^, ^36^ ^]^ MPN domain contains polar residues that resemble the active site residues of metalloproteases^[^ ^37^ ^]^ and is involved in a proteasome‐associated deneddylation activity.^[^ ^38^ ^]^ Also the MPN domain is involved in heterodimerization between CSN6 and CSN5 to regulate Cullin neddylation.^[^ ^14^ ^]^ How S60 phosphorylation participates in any activity of the MPN domain remains to be identified. However, this PKB/Akt phosphorylation site is critical to maintaining the stability of CSN6 and preserving the capability to increase the steady state expression of COP1 (Figure 7C), which in turn enhances the COP1‐mediated ubiquitination of FOXO4. Second, COP1's impact on FOXO4 ubiquitination/degradation is different from other two observations: ubiquitin ligase component Skp2 and MDM2 are two regulators for polyubiquitination of FOXO factors and mediate their degradation.^[^ ^33^, ^39^ ^]^ However, phosphorylation of FOXO proteins by PKB/Akt, IKappaB kinase (IKK) and ERK is critical for their ubiquitination mediated by SKP2 or MDM2.^[^ ^40^ ^]^ For example, Skp2 requires PKB/Akt‐specific phosphorylation of FOXO1 at Ser‐256 to have an impact.^[^ ^39^ ^]^ Our studies show that constitutively active mutant FOXO4A3,^[^ ^41^ ^]^ an PKB/Akt phosphorylation mutant of FOXO4, is still sensitive to CSN6/COP1‐mediated degradation, suggesting that CSN6/COP1‐mediated FOXO4 degradation is not similar to the action mode of Skp2 or MDM2. Because three proteins are all involved in FOXO4 ubiquitination, the potential difference between COP1 and Skp2 or MDM2 in regulating FOXO4 ubiquitination warrants further studies. Nevertheless, it makes sense that two potential oncoproteins, such as CSN6 and COP1, are involved in degrading a tumor suppressor FOXO4 to promote cell proliferation, survival, and cancer growth. COP1 is an inhibitor of p53 activity^[^ ^22^ ^]^ and thus functions like an oncoprotein. However, COP1 knockout mouse model studies suggest that COP1 may also behave like a tumor suppressor by mitigating the oncogenic activity of c‐Jun and ETS^[^ ^20^, ^21^, ^30^ ^]^ in some tissues. To resolve this discrepancy, its role in cancer needs to be further investigated. A better understanding of the COP1 regulatory complexity is a key to clarify its role in cancer. Here, we identify that COP1 diminished the steady‐state expression of FOXO4, a critical transcriptional activator involved in cell cycle and apoptosis regulation, appearing as a new E3 ligase of FOXO4.

COP1 binds VP motif on FOXO4 and facilitates FOXO4 ubiquitination, as FOXO4 (VP→AA) construct is resistant to COP1‐mediated ubiquitination and degradation, offering important insight into the structure/functional relationship between COP1 and FOXO4. Also, COP1 RING mutant has no impact on FOXO4. The fact that COP1 functions as an E3 ligase of p53, p27, and FOXO4 to accelerate their degradation,^[^ ^27^, ^28^ ^]^ manifests an oncogenic role for COP1. The oncogenic CSN6's positive impact on COP1 expression in decreasing FOXO4 stability is consistent with the concept that CSN6 and COP1 act like oncogene. The COP1 KO mouse model shows a surprising activity about COP1's tumor suppressor role. However, human cancer sample analyses indicate that COP1 is highly expressed in many types of cancer and that COP1 overexpression correlates with poor survival,^[^ ^27^, ^28^ ^]^ conflicting with the idea that COP1 can be a tumor suppressor. Our data suggest that COP1 knockout mouse cancer studies may have discrepancies that need to be resolved.

FOXO4 binds to target gene promoters to regulate the transcription of genes, but its role in regulating metabolism genes is not well characterized.^[^ ^42^ ^]^ We showed that FOXO4 binds to the promoters of SGOC pathway genes to suppress their expression. SGOC pathway is involved in methionine cycle and foliate cycle, contributing to nucleotide synthesis, methylation reactions, and the generation of NADPH for antioxidant defense.^[^ ^43^, ^44^, ^45^, ^46^, ^47^ ^]^ How FOXO4 mediates the transcriptional suppression of these genes remains to be determined. However, the FOXO4‐SGOC pathway link is identified for the first time, adding another layer of FOXO4's role in controlling cancer metabolism reprogramming.

We have shown the reverse relationship between CSN6 and FOXO4 in CRC (Figure 8). In a parallel study, we also demonstrated that high CSN6 and low FOXO4 correlate with poor survival in breast cancer (Figure S19A, Supporting Information). CSN6 and COP1 are critical for FOXO4 target gene expression (Figure S19B–D, Supporting Information). In lymphoma cells CSN6 seems to regulate FOXO target gene—Trail (Figure S19E,F, Supporting Information). Further, CSN6 reduces FOXO4 expression in breast cancer cell line (Figure S20A, Supporting Information). CSN6 regulates FOXO4‐targeted genes involved in apoptosis such as Trail (Figure S20B–D, Supporting Information). These data suggested that CSN6‐FOXO4 axis deregulation could exist in many types of cancer.

In our cancer model study, CSN6 overexpression promoted cancer growth while CSN6 knockdown inhibited tumor growth in xenograft colon cancer model. Further examination of these tumor model samples demonstrates that FOXO4 expression levels, and SGOC pathway genes are regulated according to the CSN6 knockdown. Thus, these studies recapitulated the relationship between CSN6 and FOXO4 in vivo. The studies also imply that targeting CSN6, FOXO4, or SGOC pathway could be a strategic design in inhibiting tumor growth. For example, PKB/Akt inhibitor, PHGDH inhibitor (such as a new small‐molecule PHGDH inhibitor, NCT‐503^[^ ^48^ ^]^) and/or EGFR inhibitor (Cetuximab) could be a combination for a synergistic effect (Figure S21, Supporting Information).

In summary, our data manifest a link of CSN6/COP1 regulation, FOXO4 stability, and tumorigenicity. The role of EGF/CSN6/COP1 in attenuating FOXO4 offers a new layer of regulation regarding the activity of FOXO4 in cell cycle and tumorigenicity. Further developing inhibitors that hinder EGF/CSN6/COP1‐mediated FOXO4 degradation and functions can be a strategy for rational cancer therapy.

## 4 Experimental Section

##### Cell Culture and Reagents

Human 293T, HCT116, SW480, and U2OS cells were cultured with Dulbecco's modified Eagle's medium media with 10% fetal bovine serum and antimicrobials. Human HBL1, DLD1, and BT483 cells were maintained in RPMI‐1640 medium supplemented with fetal bovine serum and antimicrobials. For transient transfection, cells were transfected with DNA using Lipofectamine 2000 (Invitrogen). Antibodies to the following epitopes and proteins were purchased from the indicated vendors: CSN6 (Enzo Life Sciences), HA (12CA5, Roche and Proteintech), ubiquitin (Zymed Laboratories), FOXO4 (Cell Signaling Technology and Santa Cruz Biotechnology) and COP1 (Bethyl Laboratories and Santa Cruz Biotechnology). Flag (M2 monoclonal antibody), PSAT1 and SHMT2 (Affinity Biosciences). PHGDH and Actin were purchased from Sigma. pPKB/Akt, PKB/Akt, and Glut1 were purchased from Cell Signaling Technology. GFP, MDM2, and Myc (mouse monoclonal 9E10) were purchased from Santa Cruz Biotechnology.

##### Plasmids

pcDNA6‐Myc‐CSN6 was constructed by PCR. CSN6 (S60A) was constructed using a site‐mutagenesis technique (Stratagene). Flag‐CSN6 was previously described.^[^ ^13^ ^]^ pCMV5‐Flag‐COP1 was kindly provided by E. Bianchi. COP1‐C136S/CS39S mutant was generated using PCR‐directed mutagenesis (Stratagene) and verified by DNA sequencing. GFP‐COP1 was constructed by PCR cloning. HA‐FOXO4 wild type and HA‐FOXO4A3 were kindly provided by Dr. Burgering. HA‐FOXO4 VPAA mutants were generated using PCR‐directed mutagenesis.

##### Immunoprecipitation and Immunoblotting

Total cell lysates were solubilized in lysis buffer (50 × 10^−3^ m Tris pH 7.5, 150 × 10^−3^ m NaCl, 1 × 10^−3^ m EDTA, 0.5% Nonidet P‐40, 0.5% Triton X‐100, 1 × 10^−3^ m phenylmethylsulfonyl fluoride, 1 × 10^−3^ m sodium fluoride, 5 × 10^−3^ m sodium orthovanadate, and 1 µg mL^−1^ each of aprotinin, leupeptin, and pepstatin) and processed as previously described.^[^ ^49^ ^]^ Lysates were immunoprecipitated with indicated antibodies. Proteins were resolved by SDS‐PAGE gels and then proteins were transferred to polyvinylidene difluoride membranes (Millipore). The membranes were blocked with 5% nonfat milk for 1 h at room temperature prior to incubation with indicated primary antibodies. Subsequently membranes were washed and incubated for 1 h at room temperature with peroxidase‐conjugated secondary antibodies (Thermo Scientific). Following several washes, chemiluminescent images of immunodetected bands on the membranes were recorded on X‐ray films using the enhanced chemiluminescence (ECL) system (Millipore).

##### In Vivo Ubiquitination Assay

HCT116 cells were used to detect endogenous FOXO4 ubiquitination. 293T cells were transiently cotransfected with indicated plasmids to detect exogenous FOXO4 ubiquitination. Forty eight hours later, cells were treated with 5 µg mL^−1^ MG132 (Sigma) for 6 h before harvesting. Cells were lysed in denaturing buffer (6 m guanidine‐HCl, 0.1 m Na_2_HPO_4_/NaH_2_PO_4_, 10 × 10^−3^ m imidazole). The cell lysates were then incubated with nickel beads for 3 h, washed, and immunoblotted with anti‐HA.

##### Protein Turnover Assay

To perform protein turnover assay, cells were transfected with the indicated plasmids and incubated at 37 °C with 5% (vol/vol) CO_2_ for 24 h. Then cycloheximide was added into the media to a final concentration of 100 µg mL^−1^. The cells were harvested at the indicated times after CHX treatment. The protein levels were analyzed by immunoblotting.

##### Targeted Metabolomics Analysis

HCT116 CSN6 overexpressing and vector control cells that were harvested and stored in an Eppendorf Safelock microcentrifuge tube, was mixed with 10 prechilled zirconium oxide beads and 20 µL of deionized water. The sample was homogenated for 3 min and 150 µL of methanol containing internal standard was added to extract the metabolites. The sample was homogenated for another 3 min and then centrifuged at 18 000 × *g* for 20 min. Then the supernatant was transferred to a 96‐well plate. The following procedures were performed on a Biomek 4000 workstation (Biomek 4000, Beckman Coulter, Inc., Brea, CA, USA). 20 µL of freshly prepared derivative reagents was added to each well. The plate was sealed and the derivatization was carried out at 30 °C for 60 min. After derivatization, the sample was evaporated for 2 h. 330 µL of ice‐cold 50% methanol solution was added to reconstitute the sample. Then the plate was stored at ‐20 °C for 20 min and followed by 4000 × *g* centrifugation at 4 °C for 30 min. 135 µL of supernatant was transferred to a new 96‐well plate with 10 µL internal standards in each well. Serial dilutions of derivatized stock standards were added to the left wells. Finally, the plate was sealed for LC‐MS analysis.

All of the standards were obtained from Sigma‐Aldrich (St. Louis, MO, USA), Steraloids Inc. (Newport, RI, USA) and TRC Chemicals (Toronto, ON, Canada). All the standards were accurately weighed and prepared in water, methanol, sodium hydroxide solution, or hydrochloric acid solution to obtain individual stock solution at a concentration of 5.0 mg mL^−1^. Appropriate amount of each stock solution was mixed to create stock calibration solutions.

A ultraperformance liquid chromatography coupled to tandem mass spectrometry (UPLC‐MS/MS) system (ACQUITY UPLC‐Xevo TQ‐S, Waters Corp., Milford, MA, USA) was used to quantitate all targeted metabolites in this study by Metabo‐Profile Biotechnology (Shanghai) Co., Ltd. The optimized instrument settings are briefly described as follows. For HPLC, column: ACQUITY HPLC BEH C18 1.7 × 10^−6^ m VanGuard precolumn (2.1 × 5 mm) and ACQUITY HPLC BEH C18 1.7 × 10^−6^ m analytical column (2.1 × 100 mm), column temp.: 40 °C, sample manager temp.: 10 °C, mobile phases: A = water with 0.1% formic acid; and B = acetonitrile/IPA (70:30), gradient conditions: 0–1 min (5% B), 1–11 min (5–78% B), 11–13.5 min (78–95% B), 13.5–14 min (95–100% B), 14–16 min (100% B), 16–16.1 min (100‐5% B), 16.1–18 min (5% B), flow rate: 0.40 mL min^−1^, and injection vol.: 5.0 µL.

For mass spectrometer, capillary: 1.5 (ESI+), 2.0 (ESI‐) Kv, source temp.: 150 °C, desolvation temp.: 550 °C, and desolvation gas flow: 1000 L h^−1^.

For data processing, the raw data files generated by UPLC‐MS/MS were processed using the MassLynx software (v4.1, Waters, Milford, MA, USA) to perform peak integration, calibration, and quantitation for each metabolite. The powerful package R studio was used for statistical analyses.

##### Untargeted Metabolomics

HCT116 CSN6‐kd and control cells were collected in 5 mL Vacutainer tubes containing the chelating agent ethylene diamine tetraacetic acid (EDTA), then the samples were centrifuged for 15 min (1500 × *g*, 4 °C). Each aliquot (150 µL) of the sample was stored at ‐80 °C until UPLC‐Q‐TOF/MS analysis. The samples were thawed at 4 °C and 100 µL aliquots were mixed with 400 µL of cold methanol/acetonitrile (1:1, v/v) to remove the protein. The mixture was centrifuged for 15 min (14 000 × *g*, 4 °C). The supernatant was dried in a vacuum centrifuge. For LC‐MS analysis, the samples were redissolved in 100 µL acetonitrile/water (1:1, v/v) solvent. To monitor the stability and repeatability of instrument analysis, quality control (QC) samples were prepared by pooling 10 µL of each sample and analyzed together with the other samples. The QC samples were inserted regularly and analyzed in every five samples.

LC‐MS/MS analyses were performed using an UHPLC (1290 Infinity LC, Agilent Technologies) coupled to a quadrupole time‐of‐flight (AB Sciex TripleTOF 6600) in Shanghai Applied Protein Technology Co., Ltd.

For HILIC separation, samples were analyzed using a 2.1 mm × 100 mm ACQUIY UPLC BEH 1.7 µm column (waters, Ireland). In both ESI positive and negative modes, the mobile phase contained *A* = 25 × 10^−3^ m ammonium acetate and 25 × 10^−3^ m ammonium hydroxide in water and B = acetonitrile. The gradient was 85% B for 1 min and was linearly reduced to 65% in 11 min, and then was reduced to 40% in 0.1 min and kept for 4 min, and then increased to 85% in 0.1 min, with a 5 min re‐equilibration period employed.

The ESI source conditions were set as follows: Ion Source Gas1 (Gas1) as 60, Ion Source Gas2 (Gas2) as 60, curtain gas (CUR) as 30, source temperature: 600 °C, IonSpray Voltage Floating (ISVF) ± 5500 V. In MS only acquisition, the instrument was set to acquire over the m/z range 60–1000 Da, and the accumulation time for TOF MS scan was set at 0.20 s per spectra. In auto MS/MS acquisition, the instrument was set to acquire over the m/z range 25–1000 Da, and the accumulation time for product ion scan was set at 0.05 s per spectra. The product ion scan is acquired using information dependent acquisition (IDA) with high sensitivity mode selected. The parameters were set as follows: the collision energy (CE) was fixed at 35 V with ± 15 eV; declustering potential (DP), 60 V (+) and −60 V (−); exclude isotopes within 4 Da, candidate ions to monitor per cycle: 10.

For data processing, the raw MS data (wiff.scan files) were converted to MzXML files using ProteoWizard MSConvert before importing into freely available XCMS software. For peak picking, the following parameters were used: centWave m/z = 25 ppm, peakwidth = c (10, 60), prefilter = c (10, 100). For peak grouping, bw = 5, mzwid = 0.025, minfrac = 0.5 were used. CAMERA (Collection of Algorithms of MEtabolite pRofile Annotation) was sued for annotation of isotopes and adducts. In the extracted ion features, only the variables having more than 50% of the nonzero measurement values in at least one group were kept. Compound identification of metabolites was performed by comparing of accuracy m/z value (<25 ppm), and MS/MS spectra with an in‐house database established with available authentic standards. After normalized to total peak intensity, the processed data were uploaded into before importing into SIMCA‐P (version 14.1, Umetrics, Umea, Sweden), where it was subjected to multivariate data analysis, including Pareto‐scaled principal component analysis (PCA) and orthogonal partial least‐squares discriminant analysis (OPLS‐DA). The sevenfold cross‐validation and response permutation testing were used to evaluate the robustness of the model. The variable importance in the projection (VIP) value of each variable in the OPLS‐DA model was calculated to indicate its contribution to the classification. Metabolites with the VIP value >1 was further applied to Student's *t*‐test at univariate level to measure the significance of each metabolite, the *p* values less than 0.05 were considered as statistically significant.

##### Extracellular Acidification Rate (ECAR) and Oxygen Consumption Rate (OCR) Measurement

Indicated CRC cells were cultured with their respectively treatments. Cells were trypsinized and plated in the XF24 microplate (Seahorse, North Billerica, MA) 8 h before the assay to have a monolayer (20 000 cells per well). Low sodium bicarbonate assay medium pH 7.4 (Seahorse, North Billerica, MA) was prepared the night before running the assay by adding 1 × 10^−3^ m sodium pyruvate, 1 × 10^−3^ m glutamine, 25 × 10^−3^ m d‐glucose and placed at 37 °C. Cartridge was rehydrated with assay buffer overnight and incubated in a hypoxic incubator. Culture media was replaced for above mentioned low sodium bicarbonate assay medium pH 7.4 and equilibrated 1 h in a hypoxic incubator at 37 °C. A series of mitochondrial inhibitors were used to determine the glycolysis and mitochondrial respiration capacities of the cells after the treatments. In order to inhibit the ATP synthase, 10 × 10^−3^ m oligomycin (Sigma, Saint Louis, MO) were added to determine mitochondrial independent oxygen consumption. To determine the maximal respiration, 10 × 10^−3^ m hydrophobic acid carbonylcyanide‐4‐(trifluoromethoxy) phenylhydrazone (FCCP) (Sigma, Saint Louis, MO), a proton ionophore was used. Finally, a combination of 10 × 10^−3^ m Rotenone (Sigma, Saint Louis, MO) and antinamycin A (Sigma, Saint Louis, MO), mitochondrial electron transport inhibitors at complex I and III respectively, were used to determine the spare respiratory capacity. Inhibitors were sequentially added to establish the ECAR and OCR. Results were analyzed using Seahorse XF software (Seahorse, North Billerica, MA). To normalize the results protein concentration was determined.

##### ChIP

ChIP was performed as described (Millipore, Catalogue # 17‐10085).

##### Glucose Uptake Assay

Glucose uptake in cells was quantified by using (2‐(N‐(7‐nitrobenz‐2‐oxa‐1,3‐diazol‐4‐yl)amino)‐2‐deoxyglucose (2‐NBDG)), a green fluorescent glucose analogue (Molecular Probes, Invitrogen). Experimental cells were incubated in glucose‐free DMEM with 10% FBS containing 120 × 10^−3^ m 2‐NBDG for time intervals ranging from 60 min. 2‐NBDG uptake was analyzed by using a fluorescent microscope (Olympus) and a FACS Canto flow cytometer (BD Biosciences). Flow cytometry data were analyzed using a FlowJo X software (FlowJo).

##### Soft Agar Colony Formation Assay

The experiment was performed as previously described.^[^ ^14^ ^]^

##### Xenograft Mouse Experiment

All animal experiments were approved by the Institutional Animal Care and Use Committee of The Sixth Affiliated Hospital of Sun Yat‐sen University (NO.20181123). HCT116 cells infected with CSN6 shRNA or control were harvested and injected into the flanks of athymic (nu/nu) female mice (6–8 weeks old). Tumor volumes were measured and recorded. At the end of the experiment, the tumors were removed and weighted.

##### Human CRC Samples and Tissue Microarray Assay

All experiments were approved by the Ethics Committee of The Sixth Affiliated Hospital of Sun Yat‐sen University (NO.2017ZSLYEC‐111). For analysis of protein expression levels of CSN6, COP1 and FOXO4, paired CRC and normal colon specimens were collected from the Department of Surgery at the Sixth Affiliated Hospital of Sun Yat‐sen University with the patients’ written informed consent and approval as previously described.^[^ ^19^, ^50^ ^]^ For TMA, paraffin‐embedded samples of primary colorectal adenocarcinomas from CRC patients were obtained. Samples were collected from the First Affiliated Hospital of Sun Yat‐sen University with the patients’ written informed consent and approval from study center's Institutional Review Board. The immunostained slides were scanned by Aperio Versa (Leica Biosystems).

##### Quantitative PCR

Total RNAs were extracted from cells using Trizol (Invitrogen); 1 µg RNA was used for producing cDNA by iScript cDNA Synthesis Kit (Bio‐Rad). Quantitative real‐time PCR analyses were performed using iQ SYBR Green Super mix (Bio‐Rad, 170‐8882) and the iCycler iQ real‐time PCR detection system. The genes’ amplification folds were analyzed relative to controls.

##### Generation of Stable Transfectants

Cells were transfected with either PCDNA6 or PCDNA6‐Myc‐CSN6 plasmids and were selected in 8 µg mL^−1^ blasticidin for 2 weeks. Cells were infected by lentiviral shRNA transduction particles (Sigma, NM_0 06833) containing either shRNA or CSN6 shRNA. After infection, cells were selected with 2 µg mL^−1^ Puromycin for 2 weeks. For generation of COP1 overexpression stable transfectants, U2OS cells were transfected with indicated for the generation of overexpression stable transfectants.

##### Luciferase Assay

A FOXO luciferase reporter gene containing a FOXO transcription factor binding site was cotransfected with the pCMV‐Myc‐CSN6 expressing vector into 293T, HCT116, or U2OS cells. Luciferase activity was assayed with the dual luciferase assay system (Promega) according to the manufacturer's instructions.

##### Lactate Dehydrogenase Activity (LDH) Assay

LDH is an oxidoreductase enzyme that catalyzes the interconversion of pyruvate and lactate. LDH activity in cells was measured by using a LDH assay kit (Sigma‐Aldrich, Catalogue #MAK066) following the manufacturer's protocol. The principle of LDH assay kit measures reduction of NAD to NADH by LDH, which is specifically detected by colorimetric (450 nm) assay. NADH was used as a standard for colorimetric detection.

##### S‐Adenosyl Methionine (SAM) Fluorescence Assay

SAM levels were measured by using a SAM fluorescence assay kit (FM‐75‐506, Mediomics). In brief, cells were lysed with buffer CM and incubated at 24 °C for 1 h, with occasional vortex. After centrifugation, the supernatant was used for SAM assay. Fluorescence signal intensity was read using a fluorescence microplate reader (excitation ≈ 485 nm, emission ≈ 665 nm).

##### FACS Analysis for Apoptosis Assay

Apoptosis was determined by two‐color analysis using propidium iodide (PI) and FITC‐conjugated anti‐Annexin V (BD Pharmingen, USA) according to the manufacturer's instructions. Cells were harvested and washed three times with PBS then cells were stained with PI and FITC‐conjugated anti‐Annexin V and analyzed with a FACSCalibur flow cytometer.

##### Cell Counting Kit‐8 (CCK8) Assay

For cell proliferation and survival assay, cells were seeded at a concentration of 1000 cells per well in 96‐well plates. Cell viability was quantified using CCK8 reagent (Dojindo Molecular Technologies) according to the manufacturer's instructions.

##### Isotope‐labeling Analysis Using LC‐MS

Metabolic analysis was performed as previously described.^[^ ^50^ ^]^ Basically, HCT116 cells were cultured in glucose free RPMI‐1640 medium, supplemented with 11 × 10^−3^ m ^13^C_6_‐glucose. After 24 h, cells were washed twice with PBS and extracted with a mixture solvent containing acetonitrile, water and formic acid (80:19:1, v/v/v). Cells were scraped, subjected to two freeze‐thaw cycles and centrifuged for 5 min at 13 000 rpm. 5 µL, 0.03 mg mL^−1^ internal standard, 4‐Cl‐phenylalanine, was added to the precipitate and then re‐extracted with methanol, and supernatants were pooled in a tube for evaporation under N2 evaporator. The dried residues were performed to a derivatization reaction using 5 (diisopropylamino)amylamine (DIAAA). The samples were mixed with 5 µL HOBt, 5 µL DIAAA‐TEA solution, and 5 µl HATU followed by 1 min incubation at room temperature. 35 µL acetonitrile was added. Samples were then analyzed by ultrahigh performance liquid chromatography‐quadrupole time‐of‐flight mass spectrometry (UHPLC‐Q‐TOF/MS), which were performed using an Agilent 1290 Infinity LC system and an Agilent 6550 UHD accurate‐mass Q‐TOF/MS system with a dual Jet stream electrospray ion source. The instrument was operated in positive ion mode and [M + H]^+^ species were measured. Data analysis was performed with the MassHunter Workstation Data Acquisition, Agilent MassHunter VistaFlux Software and Agilent Metabolite ID Software. For isotopomer labeling analysis by LC‐MS, cell pellets were lysed and protein levels were measured for normalization purposes. Targeted measurement was performed using a Dionex UltiMate 3000 LC System (Thermo Scientific) coupled to a Q Exactive Orbitrap mass spectrometer (Thermo Scientific) operated in negative mode. For calculation of the total carbon contribution in 13C‐tracing experiments, one corrected for naturally occurring isotopes (Putiande Biotechnology Corporation, Guangzhou, China). Metabolite abundance was analyzed based on the standards, MS/MS spectra, and the metabolites database METLIN (https://metlin.scripps.edu/index.php).

## Conflict of Interest

The authors declare no conflict of interest.

## Supporting information

Supporting Information
