# Corrections to EGF Relays Signals to COP1 and Facilitates FOXO4 Degradation to Promote Tumorigenesis ‐ Choi ‐ 2020 ‐ Advanced Science ‐ Wiley Online Library

**DOI:** 10.1002/advs.202508233

**Published:** 2025-07-12

https://doi.org/10.1002/advs.202000681

1) Figure 3H Western blot of actin was misplaced.


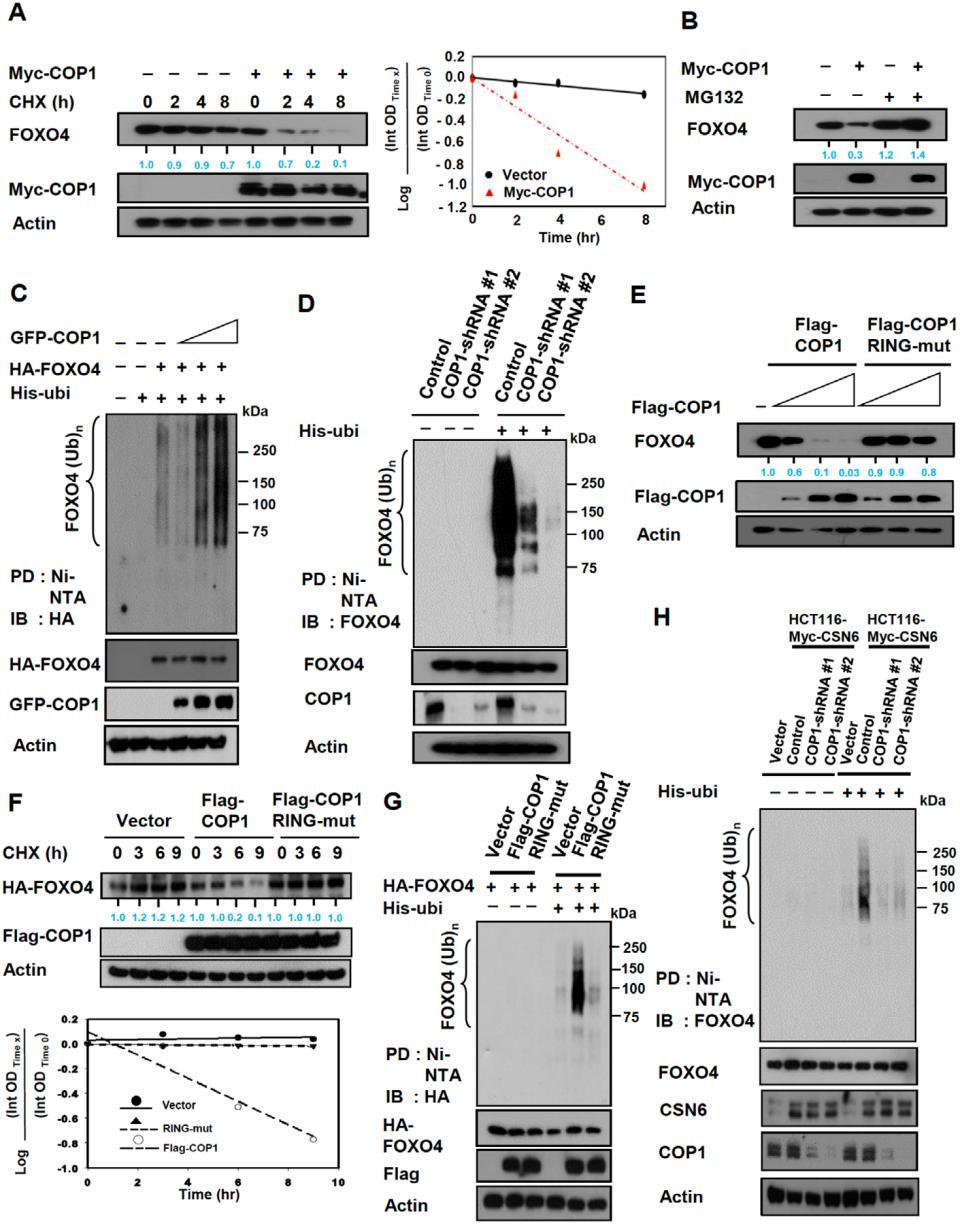


2) Figure 7D Western blot of actin was misplaced.


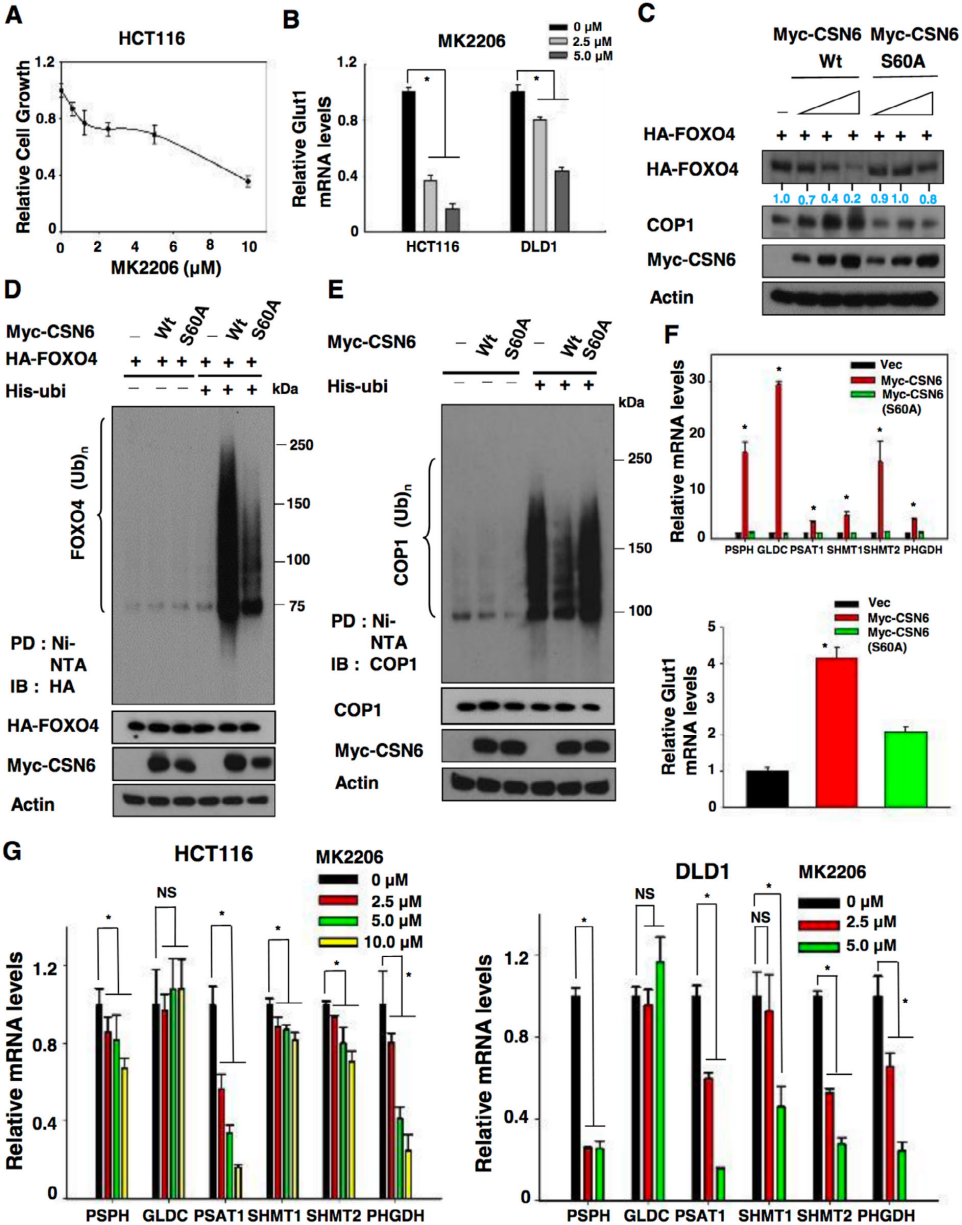


We carefully rechecked the figures and acknowledge the mistake we had made. We replaced the new actin blots. We apologize.

