# Supplementary material for: EGF Relays Signals to COP1 and Facilitates FOXO4 Degradation to Promote Tumorigenesis
Source: Adv Sci (Weinh). 2020 Sep 23;7(20):2000681. doi: 10.1002/advs.202000681 (PMC7578864; doi:10.1002/advs.202000681)
Supplement: Supplementary file 1 — Supporting Information [file ADVS-7-2000681-s001.pdf]

© 2020 Wiley-VCH GmbH

ADVANCED SCIENCE
Open Access

Supporting Information

for *Adv. Sci.,* DOI: 10.1002/advs.202000681

EGF Relays Signals to COP1 and Facilitates FOXO4 Degradation to Promote Tumorigenesis

Hyun Ho Choi, Shaomin Zou, Jian-lin Wu, Huashe Wang, Liem Phan, Kai Li, Peng Zhang, Daici Chen, Qingxin Liu, Baifu Qin, Thu Anh Thai Nguyen, Sai-Ching J. Yeung, Lekun Fang, and Mong-Hong Lee**

Supporting Information

EGF relays signals to COP1 and facilitates FOXO4 degradation to promote tumorigenesis

Hyun Ho Choi[1,2], Shaomin Zou[1,2], Jian-lin Wu[3], Huashe Wang[4], Liem Phan[5], Kai Li[1,2], Peng Zhang[1,2], Daici Chen[1,2], Qingxin Liu[1,2], Baifu Qin[1,2], Thu Anh Thai Nguyen[6], Sai-Ching J.Yeung[7], Lekun Fang[1,2,4#] and Mong-Hong Lee[1,2 #]

Supporting Information

Luciferase assay

A FOXO luciferase reporter gene containing a FOXO transcription factor binding site was co-transfected with the pCMV-Myc-CSN6 expressing vector into 293T, HCT116, or U2OS cells. Luciferase activity was assayed with the dual luciferase assay system (Promega) according to the manufacturer's instructions.

Lactate dehydrogenase activity (LDH) assay

LDH is an oxidoreductase enzyme that catalyzes the interconversion of pyruvate and lactate. LDH activity in cells was measured by using an LDH assay kit (Sigma-Aldrich, Catalogue #MAK066) following the manufacturer's protocol. The principle of LDH assay kit measures reduction of NAD to NADH by LDH, which is specifically detected by colorimetric (450 nm) assay. NADH was used as a standard for colorimetric detection.

S-Adenosyl Methionine (SAM) Fluorescence Assay

SAM levels were measured by using a SAM fluorescence assay kit (FM-75-506, Mediomics). In brief, cells were lysed with buffer CM and incubated at 24℃for 1hr, with occasional vortex. After centrifugation, the supernatant was used for SAM assay. Fluorescence signal intensity was read using a fluorescence

microplate reader (excitation ~ 485 nm, emission ~ 665 nm).

Xenograft mouse experiment

All animal experiments were approved by the Institutional Animal Care and Use Committee of The Sixth Affiliated Hospital of Sun Yat-sen University (NO.20181123). HCT116 cells infected with CSN6 shRNA or control were harvested and injected into the flanks of athymic (nu/nu) female mice (6-8 weeks old). Tumor volumes were measured and recorded. At the end of the experiment, the tumors were removed and weighted.

FACS Analysis for apoptosis assay

Apoptosis was determined by two-color analysis using propidium iodide (PI) and FITC-conjugated anti-Annexin V (BD Pharmingen, USA) according to the manufacturer's instructions. Cells were harvested and washed three times with PBS then cells were stained with PI and FITC-conjugated anti-Annexin V and analyzed with a FACSCalibur flow cytometer.

Cell Counting Kit-8 (CCK8) assay

For cell proliferation and survival assay, cells were seeded at a concentration of 1,000 cells per well in 96-well plates. Cell viability was quantified using CCK8 reagent (Dojindo Molecular Technologies) according to the manufacturer's instructions.

Cell lysates fractionated by gel filtration

HCT 116 cells were treated with EGF or no treatment for 20 minutes. Cells were lysed in lysis buffer (50 mM Tris pH 7.5, 0.5% Nonidet P-40, 0.1% Triton X-100, 150 mM NaCl, 0.1M EDTA). Superdex 200 (GE) gel filtration column was equilibrated and eluted with the buffer (0.01M phosphate buffer, 0.14M Nacl, pH.7.4). Cell lysates were loaded into column and fractionated through the column equipped with GE AKTA avant150 chromatography system at a flow rate of 0.7 ml/min. Eluants were collected 300 μl/fraction and boiled to condense concentration of the samples. Condensed fractions were resolved by SDS-PAGE, followed by immunoblotting with indicated antibodies.

In vitro binding assay

The GST-FOXO4 was kindly provided by Dr. Burgering. The plasmid construct was transformed into BL21 E.coli. Cells were lysed in the NETN buffer (0.5% NP40, 20mM Tris pH 8.0, 100mM EDTA). Lysates containing GST-FOXO4 were incubated with GST-beads (Glutathione Sepharose 4B, Amersham, 17-0756-01) overnight. The Myc-CSN6 or myc-COP1 were prepared by in vitro transcription and translation using TNT coupled system (Promega, San Luis Obispo, CA, USA). TNT products were incubated with GST-FOXO4 beads and immunoblotting was performed with indicated antibody.

In situ proximity ligation assay (PLA)

PLA was performed according to the standard commercial protocol (Sigma-Aldrich, DUO92101). The fixed HCT116 cells were blocked with the PLA blocking solution for 60 minutes prior to the application of the primary antibodies. Samples were kept in a wet chamber and incubated for overnight at 4°C. Subsequently the appropriate PLA secondary probe at a 1:5 dilution were added onto the samples and they were incubated at 37 °C for 60 minutes. Ligation mix was then applied to each of the sample to complete the ligation process at 37 °C for 30 minutes. Samples were then incubated with polymerisation mix for the amplification and incubated at 37 °C for 100 minutes. Following the incubation, samples were washed twice with 1X buffer B for 10 minutes. This was followed by one further wash with 0.01X buffer B for 1 minute. Then samples were mounted using Duolink in situ mounting medium with DAPI and the PL signal was imaged.

Supplementary Figures

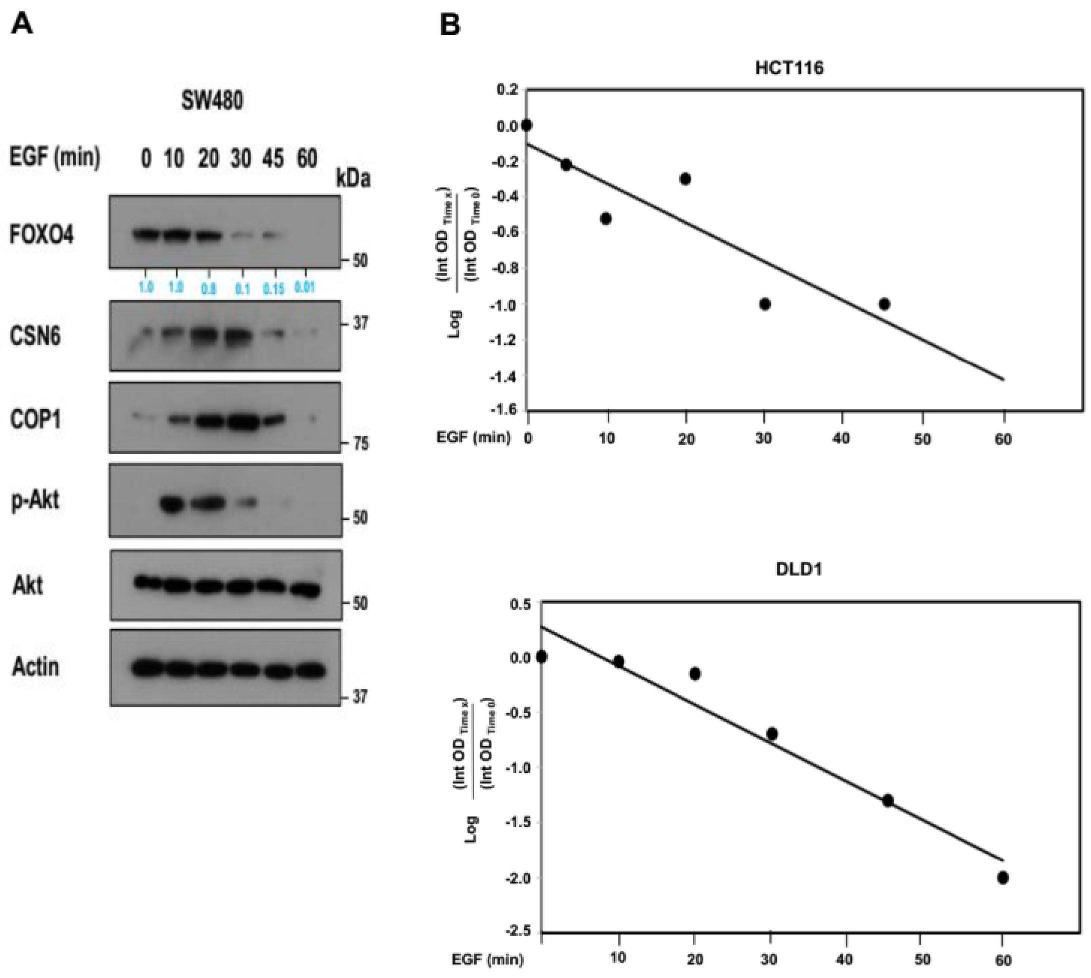

Supplementary Figure 1.

EGF treatment affects the turnover rate of FOXO4. (**A**) SW480 cells were treated with 100 ng/ml EGF for the indicated minutes. Cell lysates were analyzed by immunoblotting with indicated antibodies. (**B**) The turnover rate of FOXO4 based on the western blotting band intensity of Figure 1A is shown.

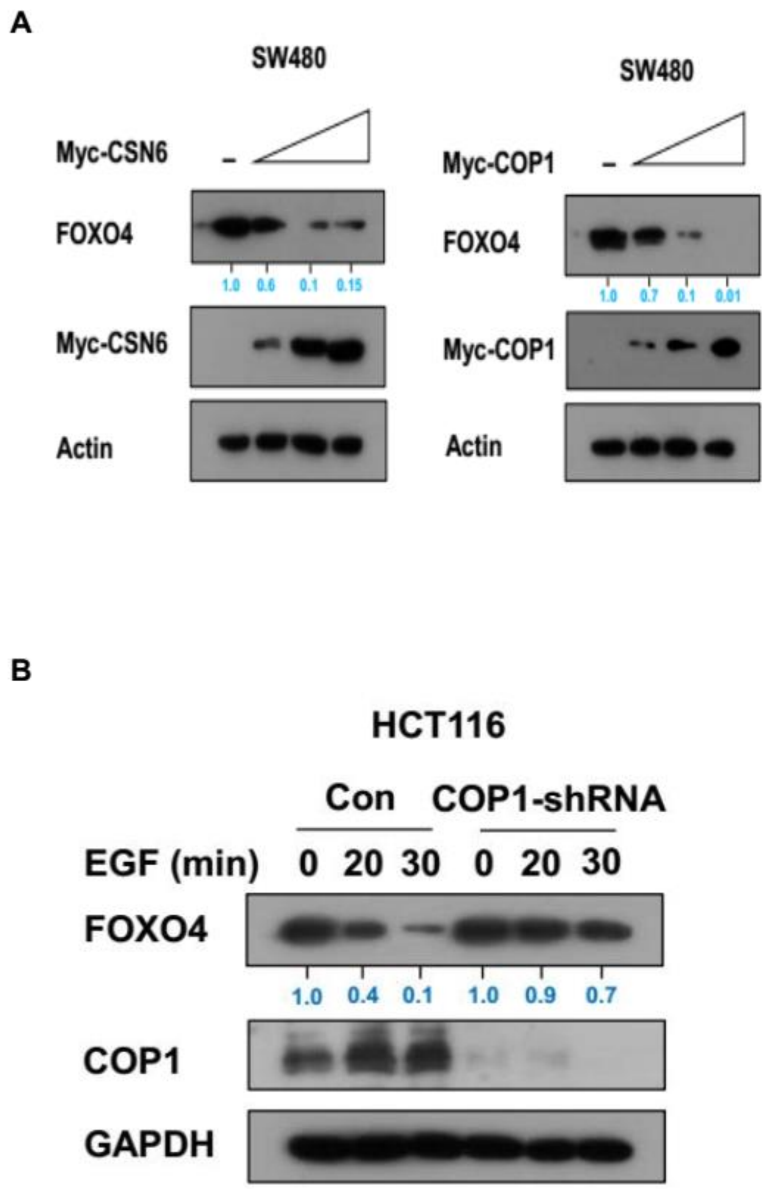

Supplementary Figure 2.

(**A**) SW480 cells were transfected with the indicated expression vector. Cell lysates were immunoblotted with indicated antibodies. (**B**) COP1 knockdown has compromised EGF-meditated turnover rate of FOXO4. HCT116 cells infected with either COP1-shRNA or control shRNA were treated with 100 ng/ml EGF for the indicated minutes. Cell lysates were immunoblotted with the indicated antibodies.

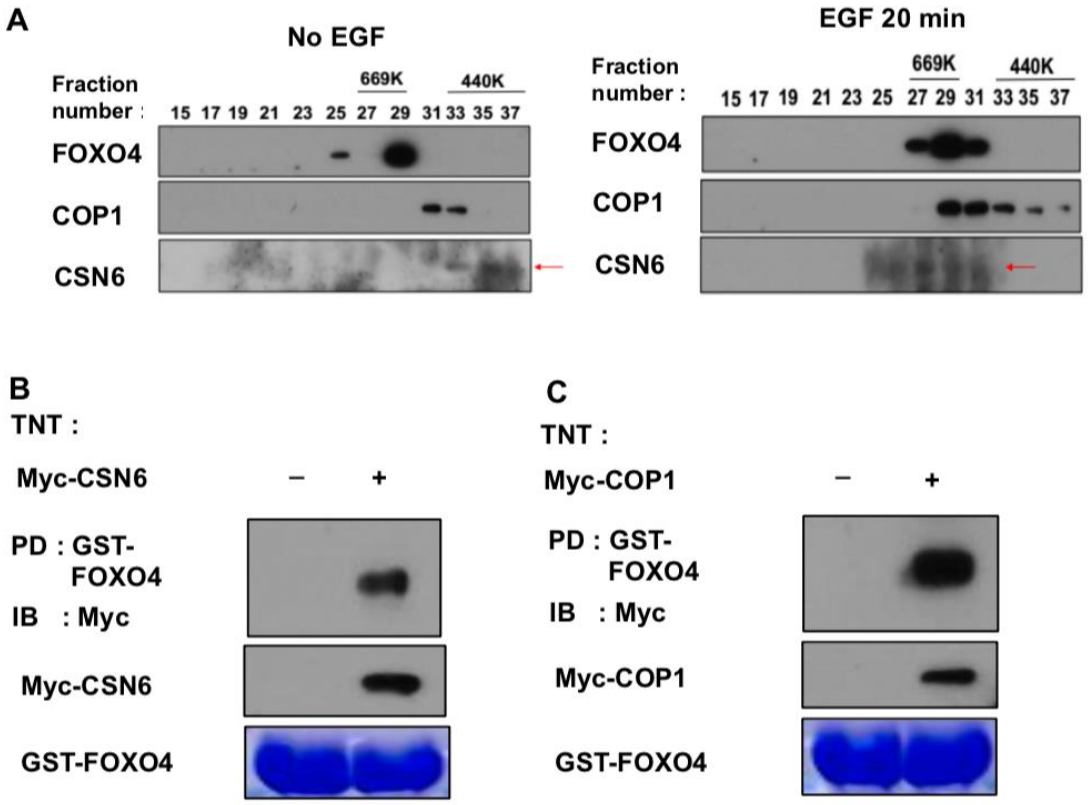

Supplementary Figure 3.

EGF stimulates CSN6/COP1-FOXO4 complex formation. (**A**) HCT 116 cells were treated with EGF or no treatment for 20 minutes. Cell lysates were prepared and analyzed for protein complex using Superdex 200 (GE) gel filtration column. Eluted fractions of protein complex were collected and analyzed by immunobloting with indicated antibodies. CSN6, COP1, and FOXO4 were coeluted at #29-31 fractions in HCT116 cells with EGF treatment for 20 minutes. GST-FOXO4 was incubated with indicated Myc-CSN6 (**B**) or Myc-COP1 (**C**), which was prepared with *in vitro* transcription & translation (TNT). Immunoblotting was performed with indicated antibodies.

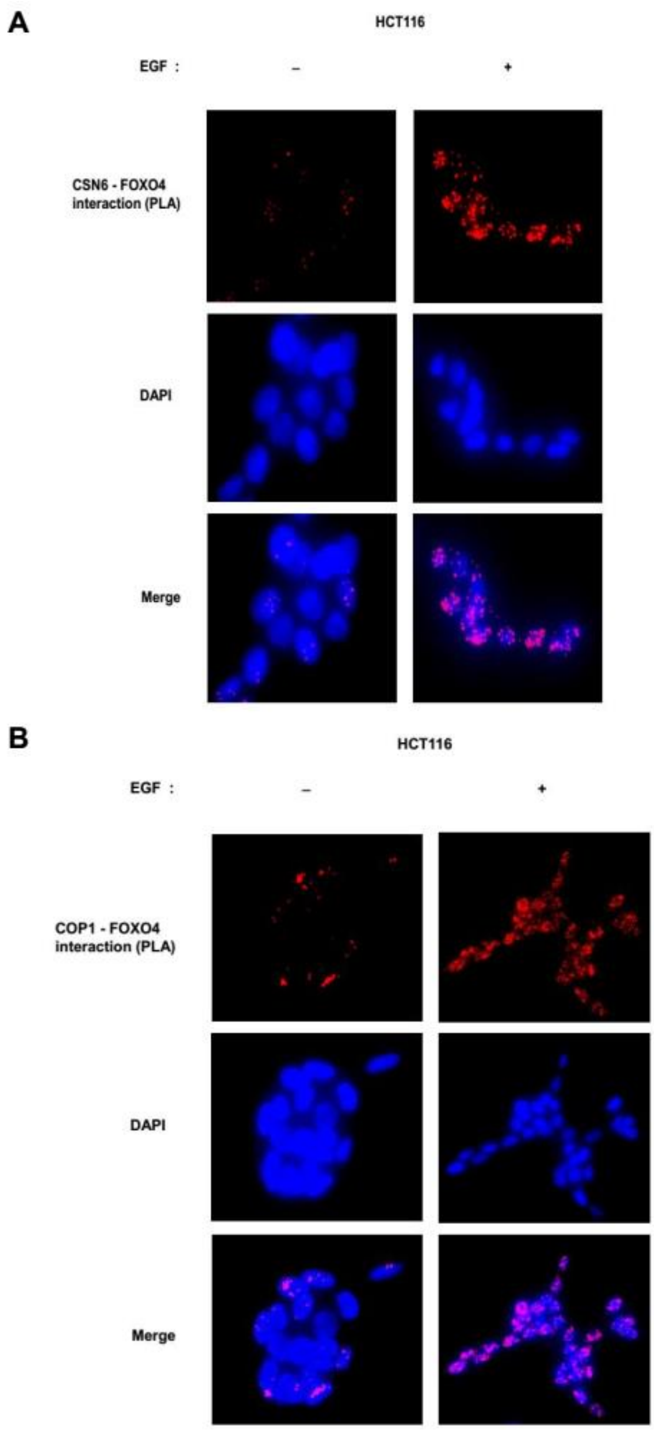

Supplementary Figure 4.

A proximity ligation assay (PLA) using antibodies against CSN6/COP1 and FOXO4. HCT116 cells were treated with 100 ng/ml EGF for 20 min. The red signals demonstrate CSN6/COP1-FOXO4 interactions based on their *in situ* proximity of 0 to 40nm. DAPI stains the nuclei of the cells.

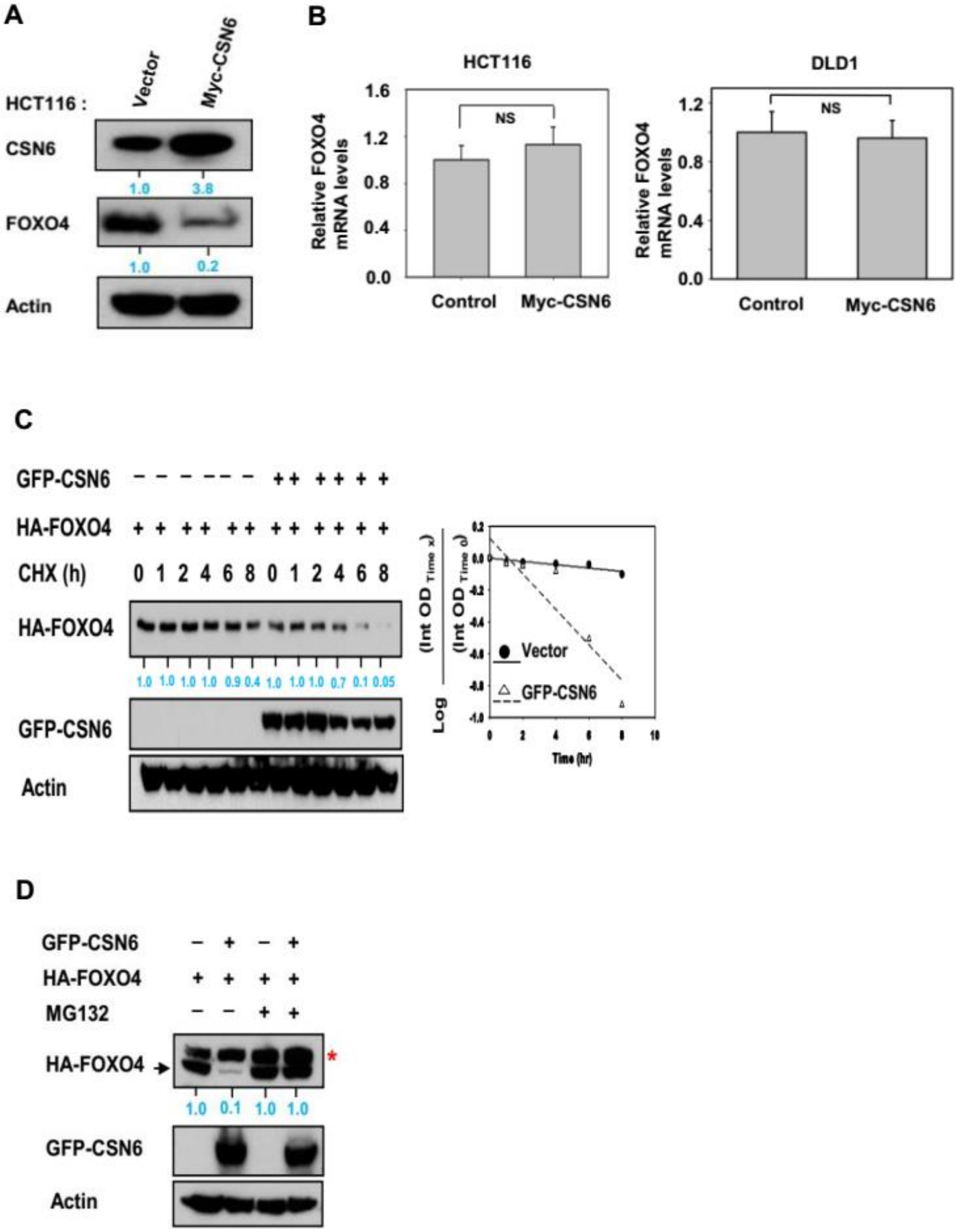

Supplementary Figure 5.

mRNA levels of FOXO4 are not affected by overexpression of CSN6. (**A**) HCT116 cells transfected with Myc-CSN6-overexpressing or vector control were immunoblotted with indicated antibodies. (**B**) Real-time quantitative PCR

analysis of FOXO4 in Myc-CSN6 and vector control transfected HCT116 or DLD1 cells. Bars represent average ± s.d., n=3, student's t-test, *P<0.05. (**C**) 293T cells transfected with the indicated plasmids were treated with cycloheximide (CHX) (100 μg/ml) for the indicated hours. Cell lysates were immunoblotted with indicated antibodies. The turnover rate of FOXO4 is shown. (**D**) 293T cells transfected with indicated plasmids were treated with or without proteasome inhibitor MG132. Lysates were immunoblotted with indicated antibodies. * indicates non-specific bands.

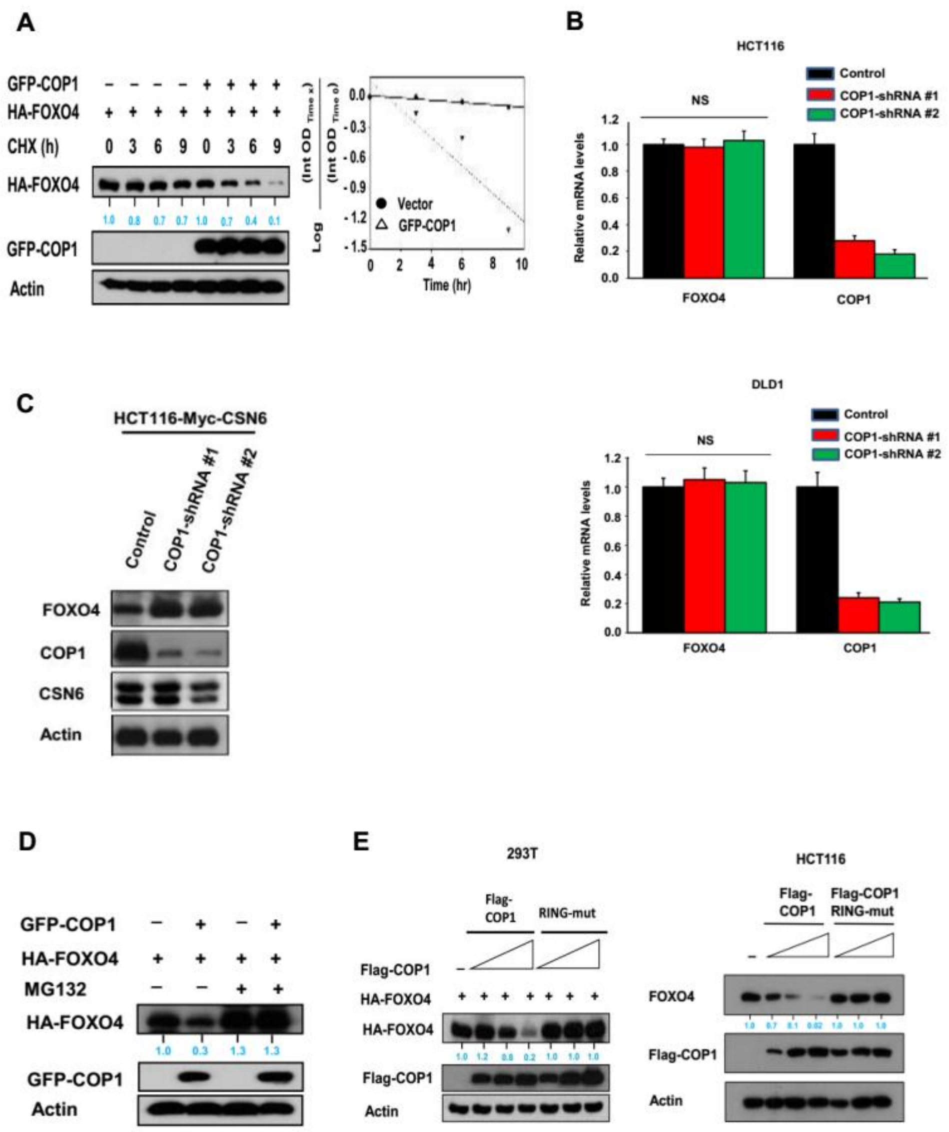

Supplementary Figure 6.

(**A**) 293T cells transfected with the indicated plasmids were treated with cycloheximide (CHX) (100 μg/ml) for the indicated hours. Cell lysates were immunoblotted with indicated antibodies. The turnover rate of FOXO4 is shown. (**B**) mRNA levels of FOXO4 are not affected by knockdown of COP1. Real-time quantitative PCR analysis of FOXO4 in HCT116 (top) or DLD1 (bottom) cells infected with either COP1 shRNA or control shRNA. Bars represent average ±

s.d., n=3, one-way ANOVA. (**C**) Lysates of Myc-CSN6-overexpressing HCT116 cells infected with either COP1 shRNA or control shRNA were immunoblotted with indicated antibodies. (**D**) 293T cells transfected with indicated plasmids were treated with or without proteasome inhibitor MG132. Lysates were immunoblotted with indicated antibodies. (**E**) 293T (left) and HCT116 (right) cells were transfected with the indicated expression vectors. Lysates were immunoblotted with indicated antibodies.

A

***Homo sapiens* FOXO4**

135
AQIYEWMVRT**VP**YFKDKGDSNS

246
PASVSSYAGG**VP**PTLNEGLELL

428
PLEAPGPSSL**VP**TLSMIAPPPV

B

Homo sapiens	***FOXO4***	PLEAPGPSSL**VP**TLSMIAPPPV
Mus musculus	***FOXO4***	PLEGPGPSNL**VP**NLSVMAPPPV
Rattus norvegicus	***FOXO4***	PLEGPGPSNL**VP**TLSVMAPPPV
Sus scrofa	***FOXO4***	PLEAPGPSSL**VP**TLPMMAPAPPPV
Callithrix jacchus	***FOXO4***	PLEAPGPSSL**VP**NPSMIAPPPV
Pan troglodytes	***FOXO4***	PLEAPGPSSL**VP**TLSMIAPPPV
Macaca mulatta	***FOXO4***	PLEAPGPSSL**VP**TLSMIAPPPV
Bos taurus	***FOXO4***	PLEAAGPSGL**VP**TLPMIAPAPPPV
Arabidopsis thaliana	***HY5***	GIESDEEIRR**VP**EFGGEAVGKE
Arabidopsis thaliana	***STH***	RYDDEEEHFL**VP**DLG
Homo sapiens	***c-JUN***	LQALKEEPQT**VP**EMPGETPPLS

Supplementary Figure 7.

FOXO4 has the VP motif for COP1 binding. (**A**)Three potential VP motifs (135VP, 296VP, 428VP) are present in human FOXO4. (**B**) Consensus COP1 binding motif, highlighted in sequences of FOXO4 428 VP motif from various species.

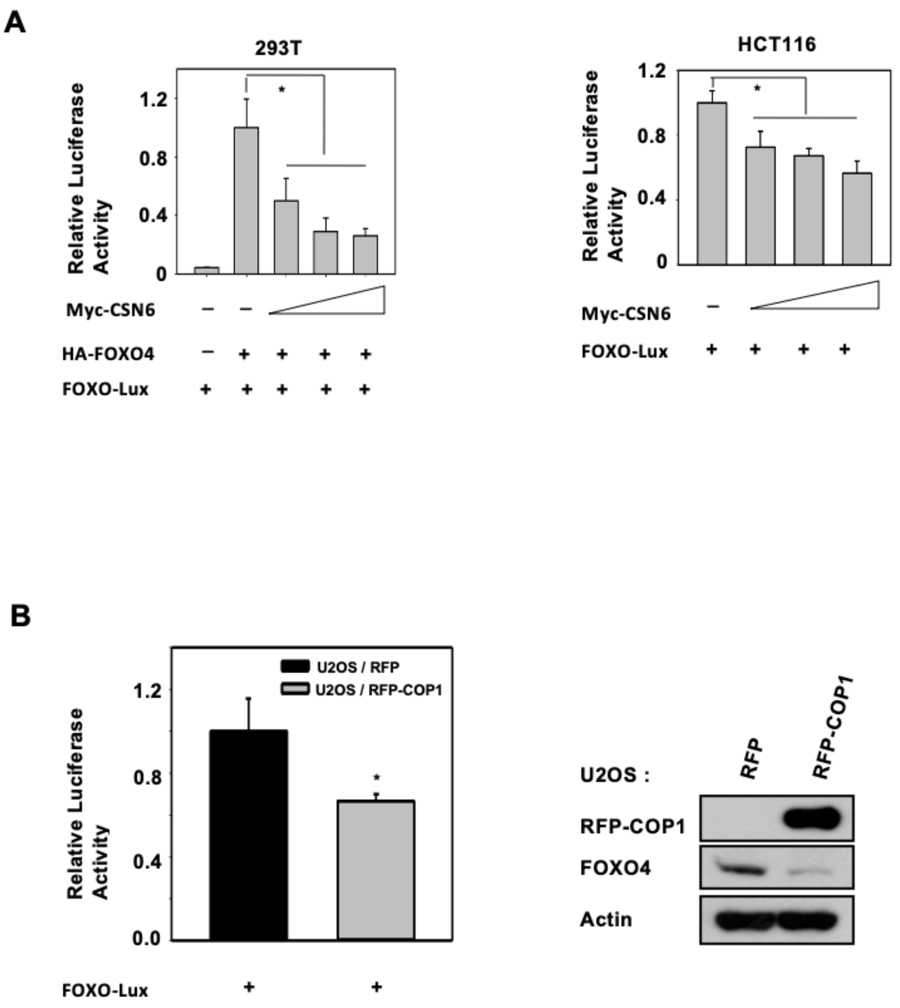

Supplementary Figure 8.

CSN6 diminishes FOXO4 transcriptional activity. (**A**) CSN6 suppresses FOXO transcriptional activity. 293T and HCT116 cells were transfected with the indicated expression vectors. FOXO transcriptional activity was determined by FOXO-responsive luciferase reporter activity. Bars represent average ± s.d., n=3, one-way ANOVA, *P<0.05. (**B**) U2OS cells were transfected with the RFP-COP1. FOXO transcriptional activity was determined by FOXO-responsive luciferase reporter activity. COP1's impact on the protein expression level of FOXO4 is shown. Bars represent average ± s.d., n=3, student's t-test, *P<0.05.

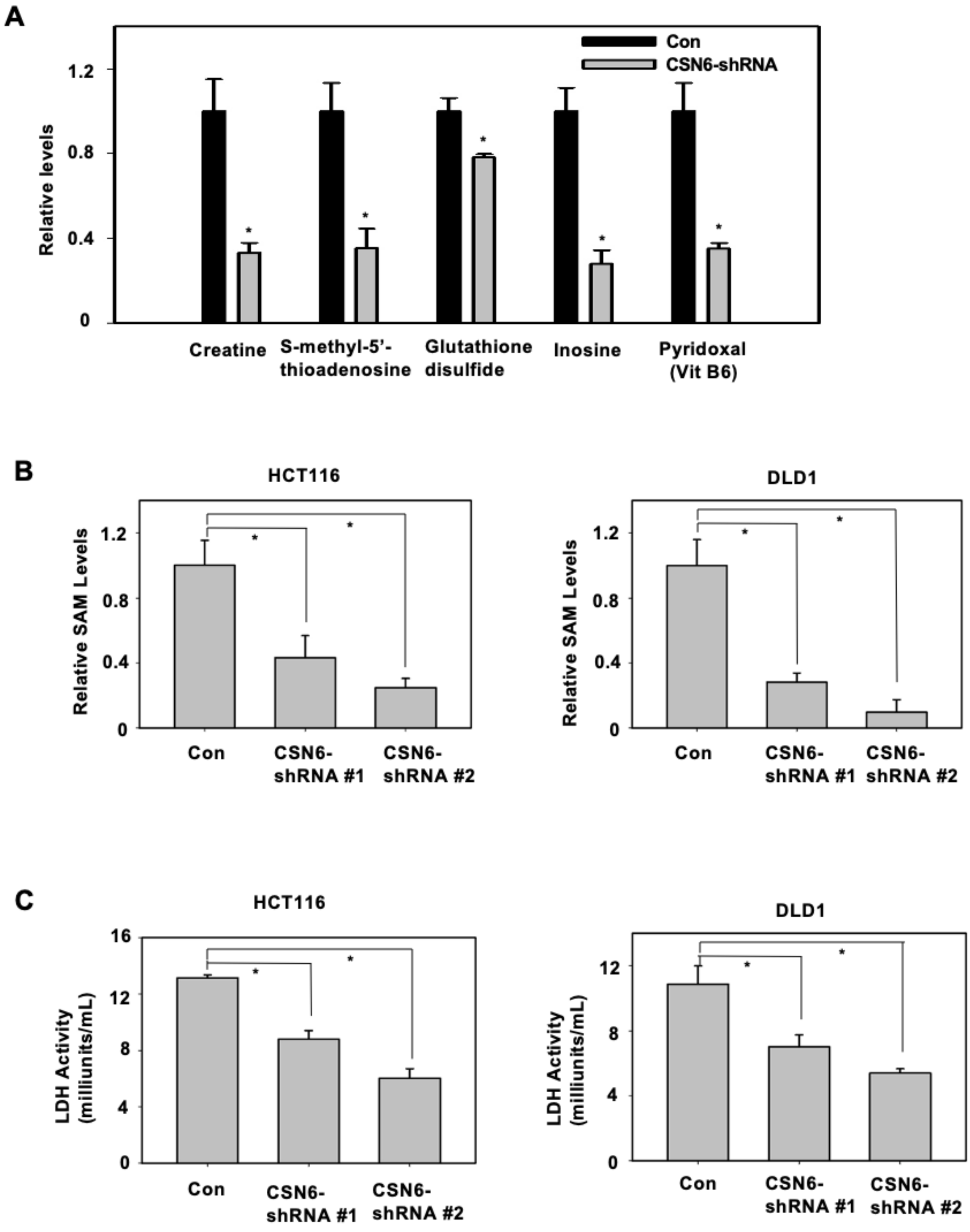

Supplementary Figure 9.

CSN6 regulates cell metabolism. (**A**) Knockdown of CSN6 reduces production of SGOC pathway related metabolites. HCT116 cells infected with control shRNA or CSN6 shRNA were used for metabolic analysis. Bars represent

average ± s.d., n=7, two-tailed Student's t-test, **P*<0.05. (**B**) Knockdown of CSN6 inhibits SAM levels in HVT116 (left) and DLD1 (right) cells. Bars represent average ± s.d., n=3, One-way ANOVA, **P*<0.05. (**C**) Knockdown of CSN6 suppresses LDH activity in HCT116 and DLD1 cells. Bars represent average ± s.d., n=3, One-way ANOVA, **P*<0.05.

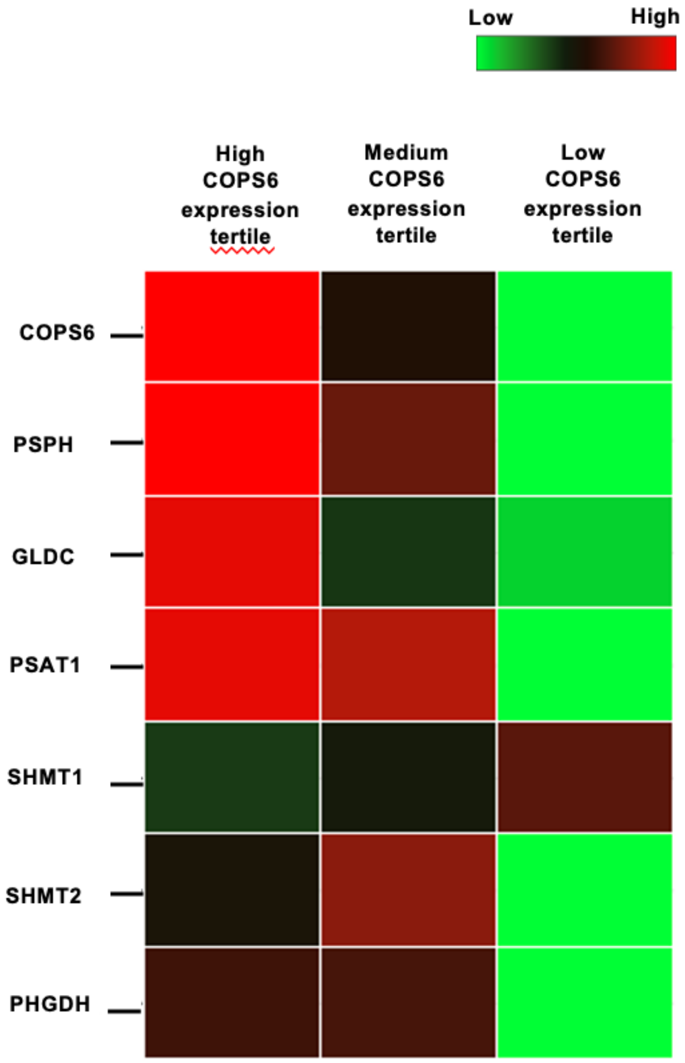

Supplementary Figure 10.

Heatmap of genes related to SGOC in colorectal cancer (GSE21510) based on COPS6 (CSN6) expression.

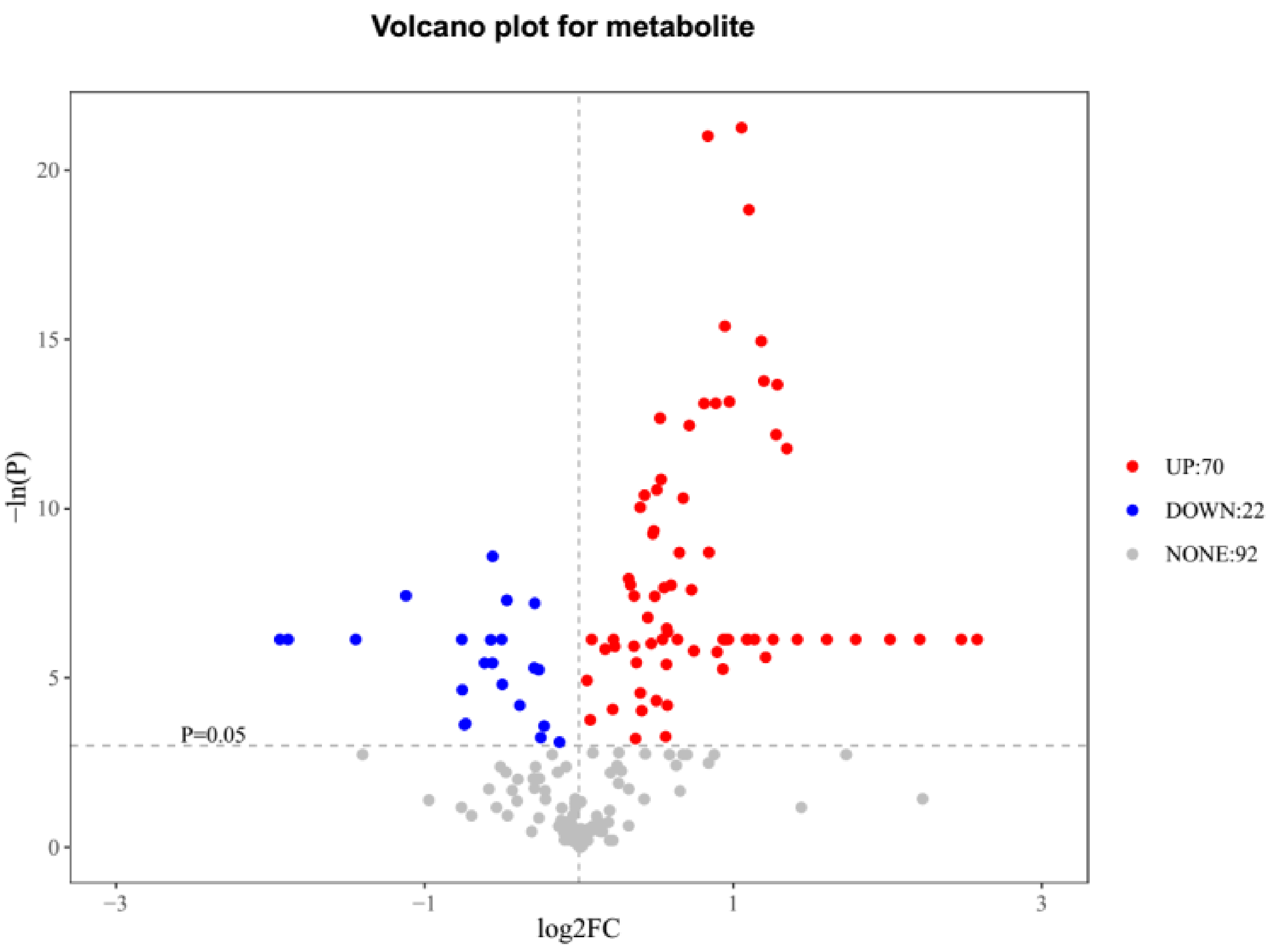

Supplementary Figure 11.

Different expression of metabolite concentrations is shown by volcano plot.

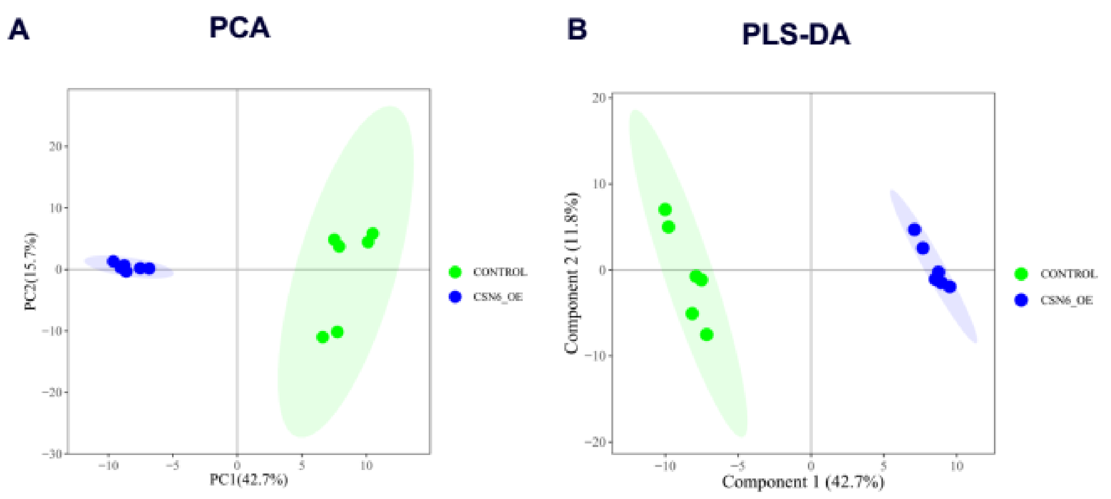

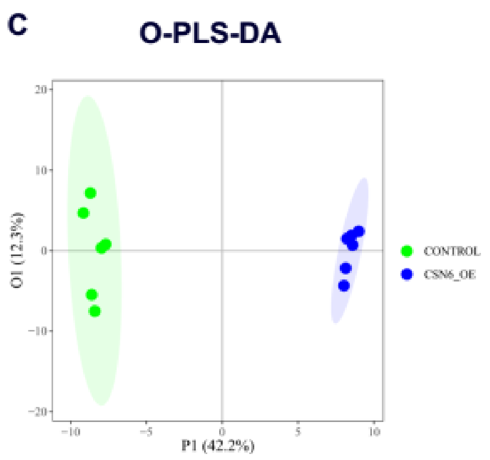

D **R2,Q2**

Type	N	R2X	R2Y	Q2
PCA	12	0.427		
PLS-DA	12	0.427	0.987	0.953
OPLS-DA	12	0.422	0.987	0.955

Supplementary Figure 12.

Principal component analysis (PCA), partial least-squares-discriminant analysis (PLS-DA), and orthogonal partial squares discriminant analysis (OPLS-DA) including proper cross-validation (R2, Q2) were performed. The significance of the changes in metabolite levels between HCT116 vector control and CSN6 overexpressing (OE) cells were assessed.

Volcano plot of negative model for metabolite

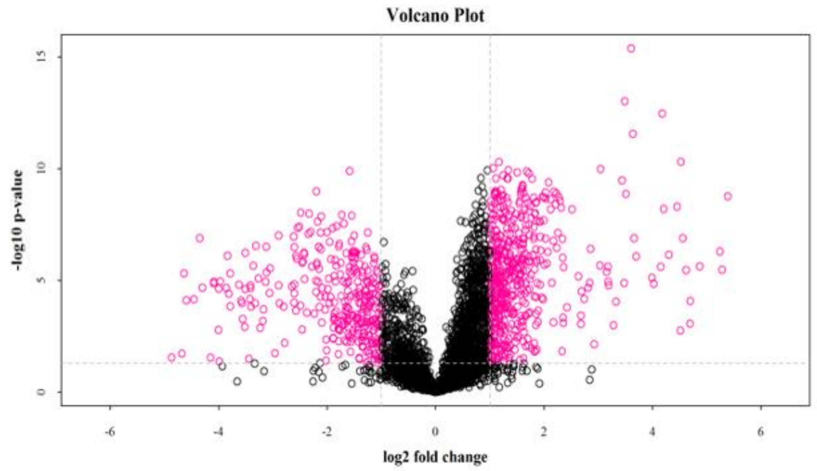

Volcano plot of positive model for metabolite

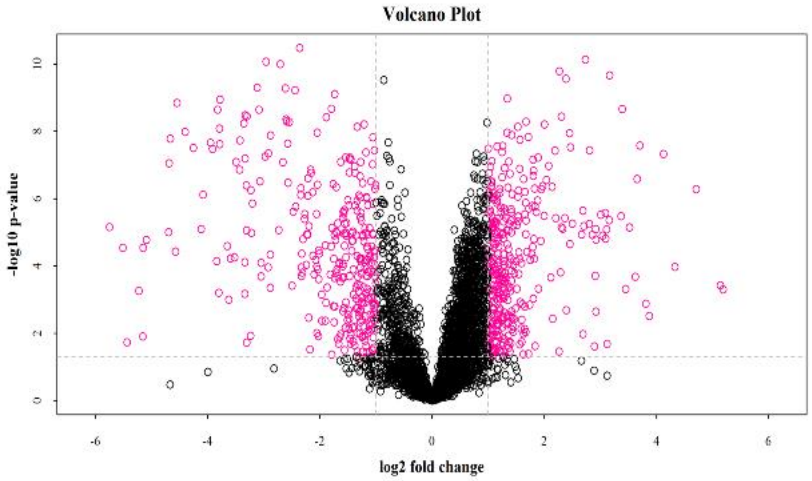

Supplementary Figure 13.

Different expression of metabolite concentrations is shown by volcano plot. Pink spots represent metabolites with Fold Change>2.0 or <0.5, and P value <0.05.

A **PCA** **O-PLS-DA**

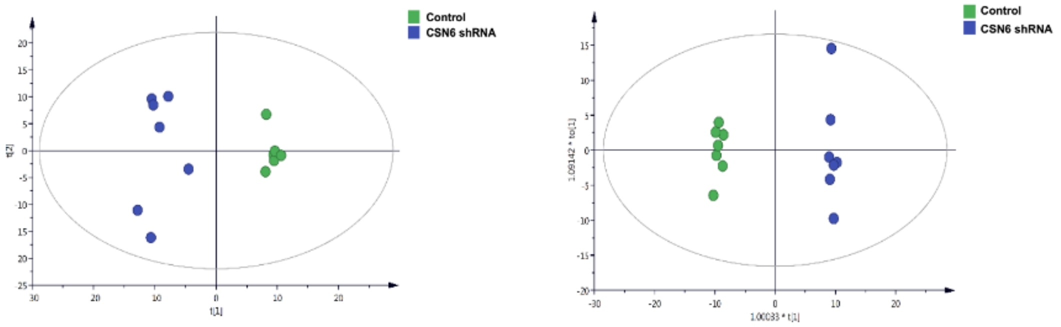

B **R2,Q2**

POS Models				
Type	N	R2X	R2Y	Q2
PCA-X	14	0. 394		0. 0403
PLS-DA	14	0. 326	0. 997	0. 867
OPLS-DA	14	0. 326	0. 997	0. 898

NEG Models				
Type	N	R2X	R2Y	Q2
PCA-X	14	0. 394		0. 0403
PLS-DA	14	0. 326	0. 997	0. 867
OPLS-DA	14	0. 326	0. 997	0. 898

Supplementary Figure 14.

Principal component analysis (PCA) and partial least-squares-discriminant analysis (PLS-DA) including proper cross-validation (R2, Q2) were performed. The significance of the changes in metabolite levels between HCT116 control and CSN6-shRNA knockdown cells were assessed.

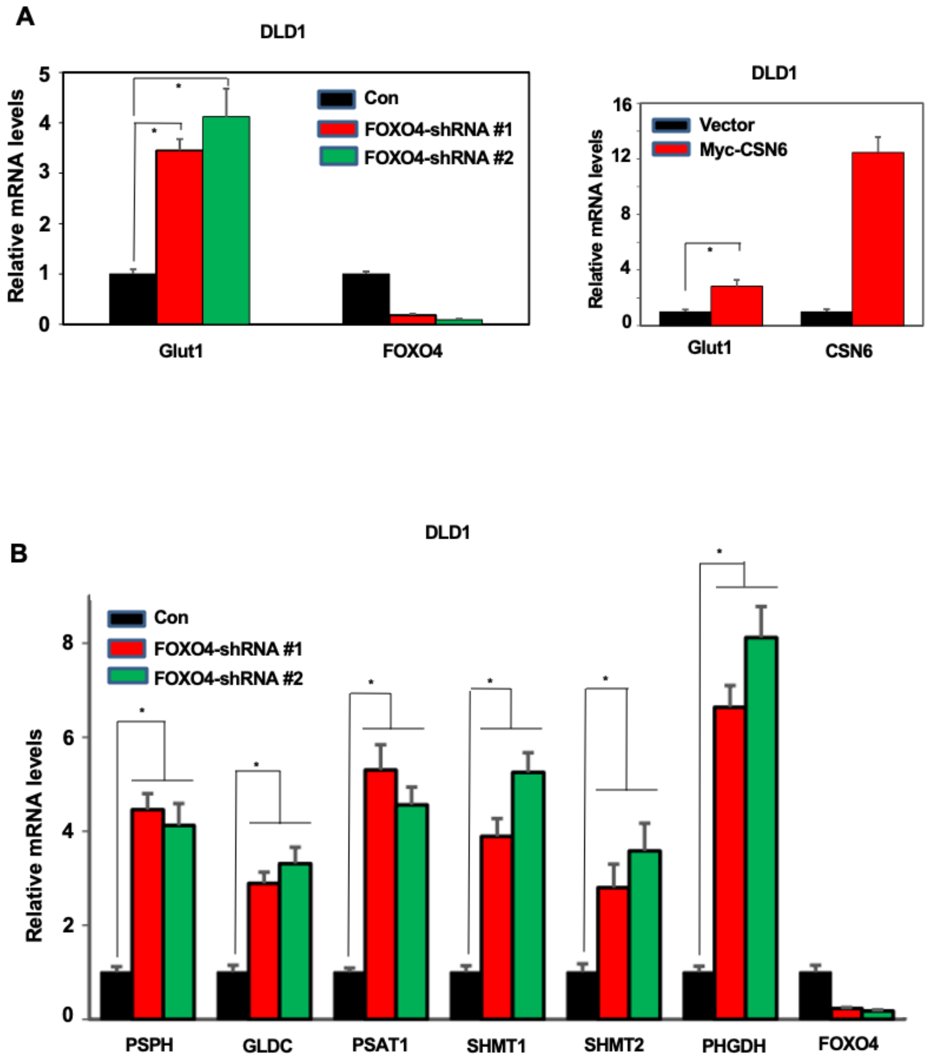

Supplementary Figure 15. Impact of FOXO4 on the expression of Glut1 and serine pathway genes. (**A**) RT-qPCR analysis of Glut1 in FOXO4 shRNA infected DLD1 cells. Bars represent average ± s.d., n=3, one-way ANOVA, **P*<0.05 (left). RT-qPCR analysis of Glut1 in Myc-CSN6 expressing DLD1 cells. Bars represent average ± s.d., n=3, student's t-test, **P*<0.05 (right). (**B**) Real-time quantitative PCR analysis of serine pathway genes in FOXO4 knockdown DLD1 cells. Bars represent average ± s.d., n=3, two-tailed Student's t-test, **P*<0.05.

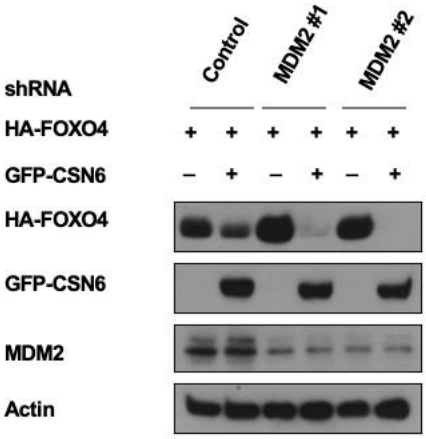

Supplementary Figure 16.

CSN6-mediated FOXO4 down regulation does not involve MDM2. 293T cells co-transfected with the indicated plasmids and infected with either MDM2 shRNA or control shRNA were immunoblotted with indicated antibodies.

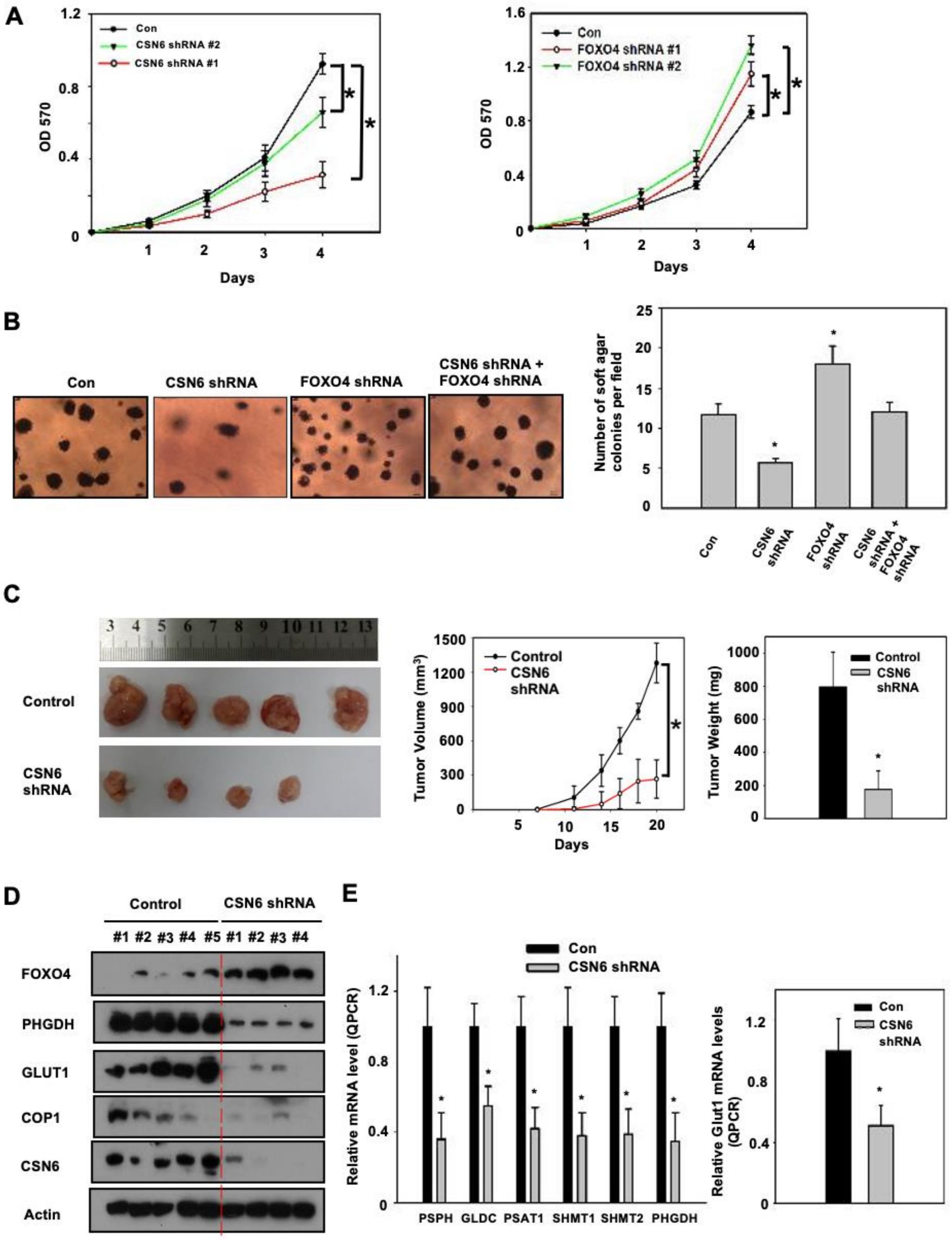

Supplementary Figure 17.

CSN6-FOXO4 axis is critical for tumorigenicity. (**A**) Cell proliferation of HCT116 cells infected with CSN6 shRNA or control was assayed by CCK8 cell counting kit (left). Cells proliferation of FOXO4 knockdown HCT116 cells were demonstrated (right). Error bars, 95% confidence interval. Values represent average ± s.d., n=8, two-tailed t test, *P<0.05. (**B**) CSN6 knockdown and

CSN6/FOXO4 double knockdown HCT116 cells were analyzed for soft agar colony formation (left). Average numbers of colonies per field were quantitated (right). Bars represent average ± s.d., n=5, one-way ANOVA, *P<0.05. (**C**) HCT116 cells infected with CSN6 shRNA or control vector were subcutaneously injected into the flank of nude mice. Representative tumors from each group are shown (left). Tumor volumes were monitored for 20 days (middle); error bars represent 95% confidence intervals. Tumor weights from each group were measured (right). Error bars represent 95% confidence intervals. Values represent average ± s.d., n=5, ANOVA (middle) and student's t-test (right), *P<0.05. (**D**) Protein expression levels of tumor lysates generated in c were analyzed with indicated antibodies. (**E**) Real-time quantitative PCR analysis of serine pathway genes (left) and Glut1 (right) in tumors generated in c. Bars represent average ± s.d., n=3, two-tailed student's t-test (middle) and student's t-test (right), *P<0.05.

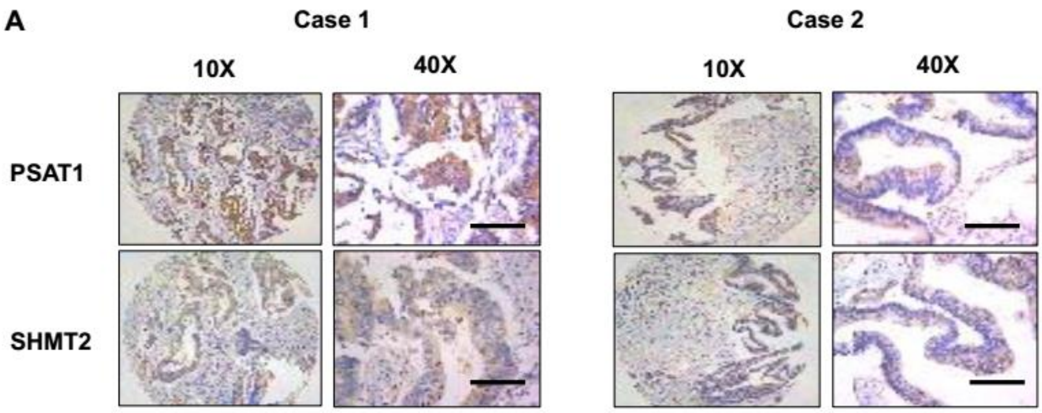

B

Human colorectal tumor (TMA)

	CSN6 low (N = 100)	CSN6 high (N = 144)	*P* value
PSAT1			0.002
Low	62 (25.4%)	59 (24.2%)	
High	38 (15.6%)	85 (34.8%)	
SHMT2			< 0.001
Low	77 (31.6%)	36 (14.8%)	
High	23 (9.4%)	108 (44.3%)	

Supplementary Figure 18.

Validation of CSN6-FOXO4 deregulation in human colorectal cancer samples.

(**A**) Representative IHC staining for PSAT1 and SHMT2 in human TMAs. Case 1 is a representative of a patient with CSN6-high colon cancer. Case 2 is a representative of a patient with CSN6-low colon cancer. Scale bar=100μm

(**B**) Quantification of staining intensities of indicated protein from sections in (A).

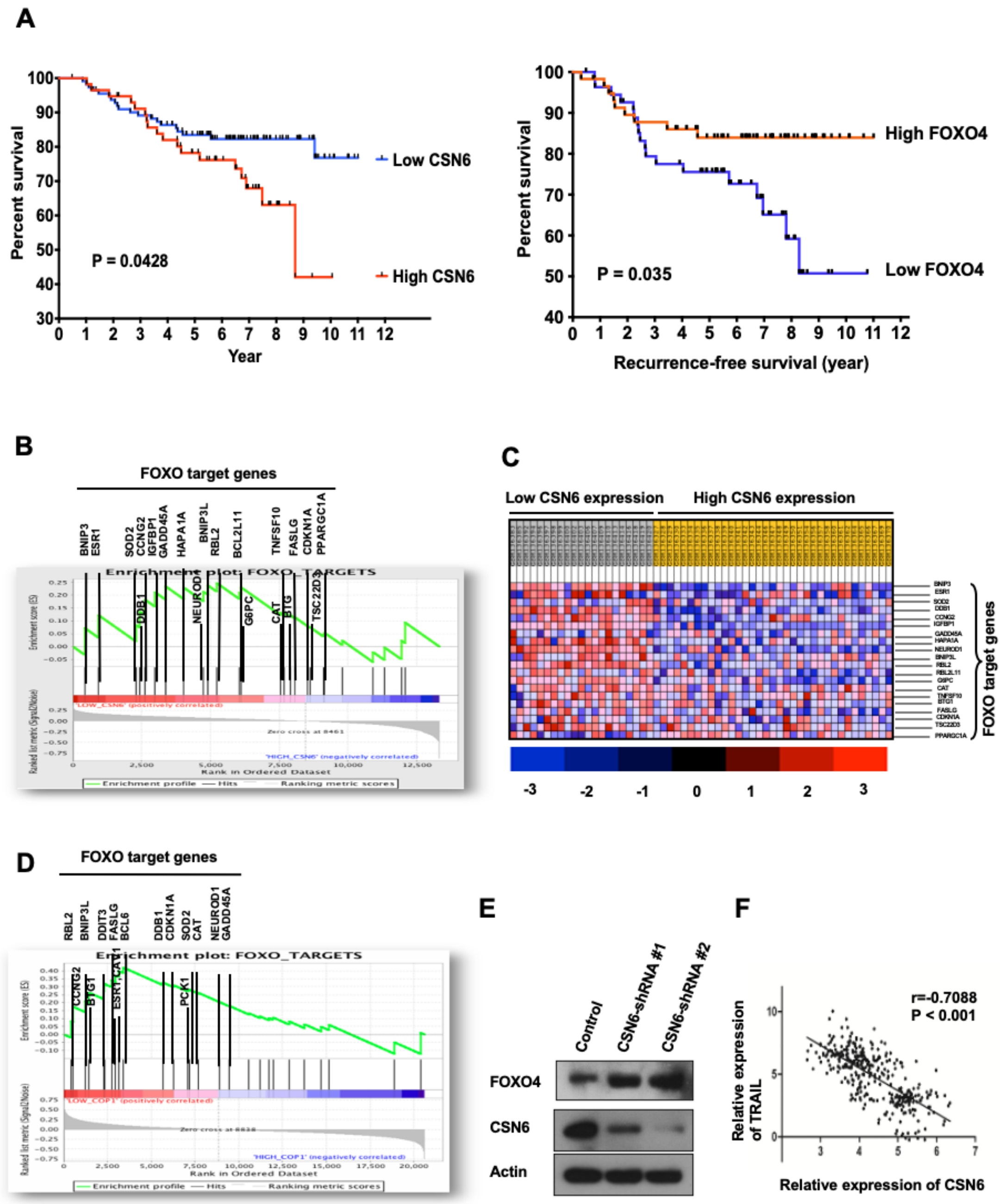

Supplemental Figure 19.

The clinical relevance of CSN6 expression in breast cancer. (**A**) The impact of CSN6 on recurrence-free breast cancer patient survival. Gene expression profiles of 225 breast cancer patients (stage I-III) were downloaded from the Gene Expression Omnibus database (cohort GSE20194), analyzed with Nexus Expression 3.0 (BioDiscovery) and matched with corresponding clinical data.

The Kaplan-Meier survival curve was built using GraphPad Prism 6.0. Log-rank (Mantel-Cox) test was used in this survival analysis (left). The impact of FOXO4 on recurrence-free breast cancer patient survival. Gene expression profiles of 225 breast cancer patients (stage I-III) were downloaded from the Gene Expression Omnibus database (cohort GSE20194), analyzed with Nexus Expression 3.0 (BioDiscovery) and matched with corresponding clinical data. The Kaplan-Meier survival curve was built using GraphPad Prism 7.0. Log-rank (Mantel-Cox) test was used in this survival analysis (right). (**B-C**) Gene Set Enrichment Analysis (GSEA, Broad Institute) of breast cancer patients showed upregulation of FOXO target genes in breast tumors that expressed a low level of CSN6 compared with breast tumors with high CSN6 expression. Heat map of FOXO target gene expression on the basis of CSN6 expression level were shown in (C) panel. (**D**) Gene Set Enrichment Analysis (GSEA, Broad Institute) of breast cancer patients showed upregulation of FOXO target genes in breast tumors that expressed a low level of COP1 compared with breast tumors with high COP1 expression. (**E**) Protein levels of FOXO4 were upregulated when endogenous COP1 expression was inhibited with shRNA in lymphoma cells. Lysates of indicated lymphoma cells (HBL1) infected with either CSN6-shRNA or control shRNA were analyzed by IB with the indicated antibodies. (**F**) Relative expression of CSN6 and TRAIL (a target gene of FOXO4) were calculated in a lymphoma data set (GSE2350). Spearman rank correlation was used to demonstrate the correlation between CSN6 and TRAIL.

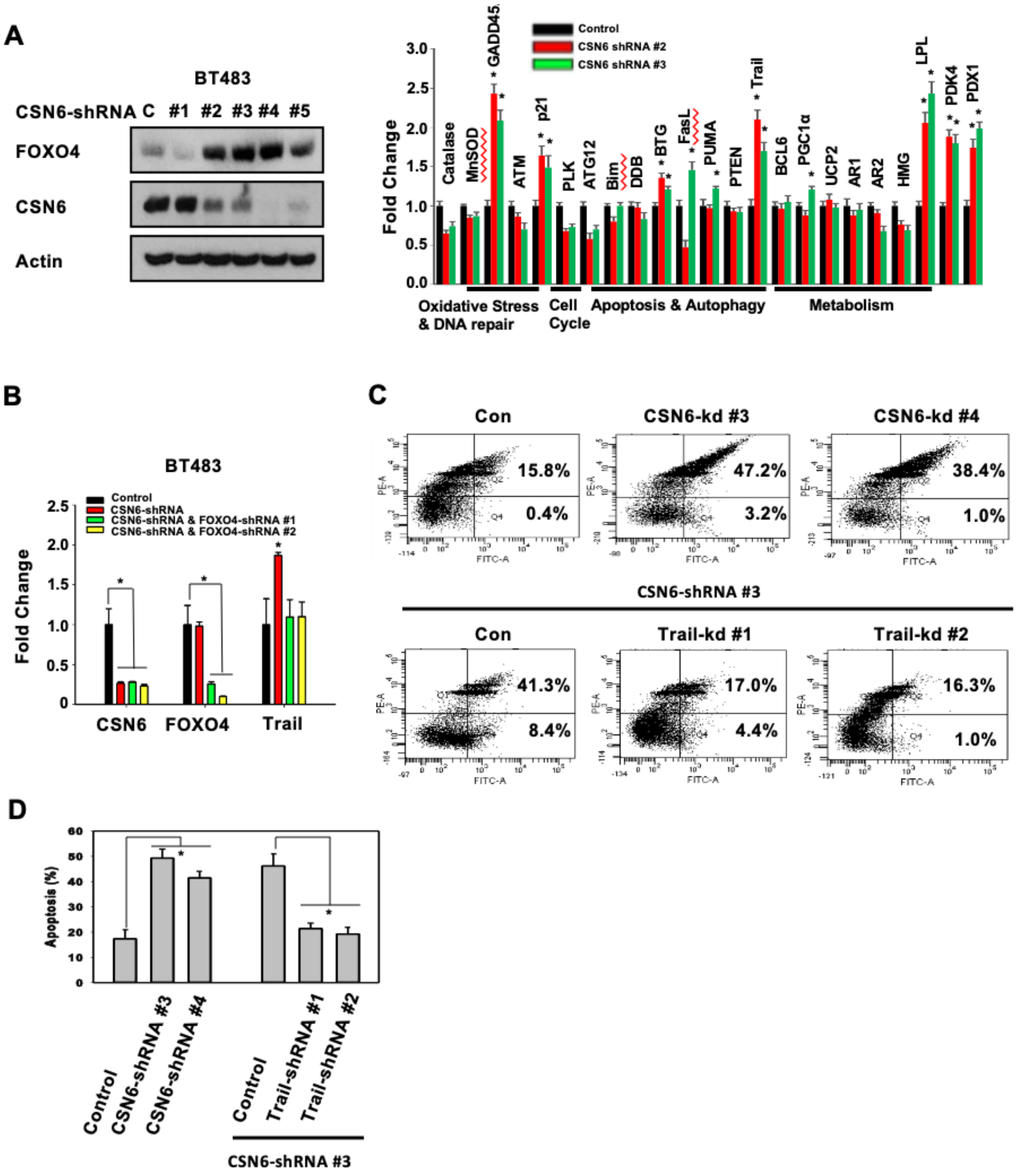

Supplementary Figure 20.

CSN6-FOXO4 axis has impact on Trail-mediated cell apoptosis. (**A**) CSN6 regulates FOXO target genes expression. Lysates of BT483 cells infected with either CSN6 shRNA or control shRNA were immunoblotted with indicated antibodies (left). mRNA levels of the indicated FOXO target genes were determined by quantitative RT-PCR in BT483 cells infected with either CSN6 shRNA or control shRNA (right). Bars represent average ± s.d., n=3, ANOVA,

*P<0.05.

(**B**) Knockdown of FOXO4 in CSN6 knockdown cells reverses CSN6 knockdown-mediated Trail up-regulation. Real-time quantitative PCR analysis of Trail mRNA levels in BT483 cells infected CSN6 shRNA or control shRNA and double knockdown cells are shown. Bars represent average ± s.d., n=3, ANOVA, *P<0.05. (**C**) Knockdown of CSN6 accelerates apoptosis induced by serum starvation. BT483 cells infected with CSN6 shRNA or control shRNA and double knockdown cells were cultured in 0% fetal bovine serum for 4 days. Binding of Annexin V and uptake of propidium iodide were analyzed by flow cytometry. The lower left quadrant contains the viable population of cells, the lower right quadrant contains early apoptotic cells, the upper left quadrant contains necrotic cells and the upper right quadrant contains late apoptotic cells. (**D**) The mean of three data sets was taken and the apoptosis is analyzed from the corresponding quadrant. Error bars represent 95% confidence intervals. Bars represent average ± s.d., n=3, ANOVA, *P<0.05.

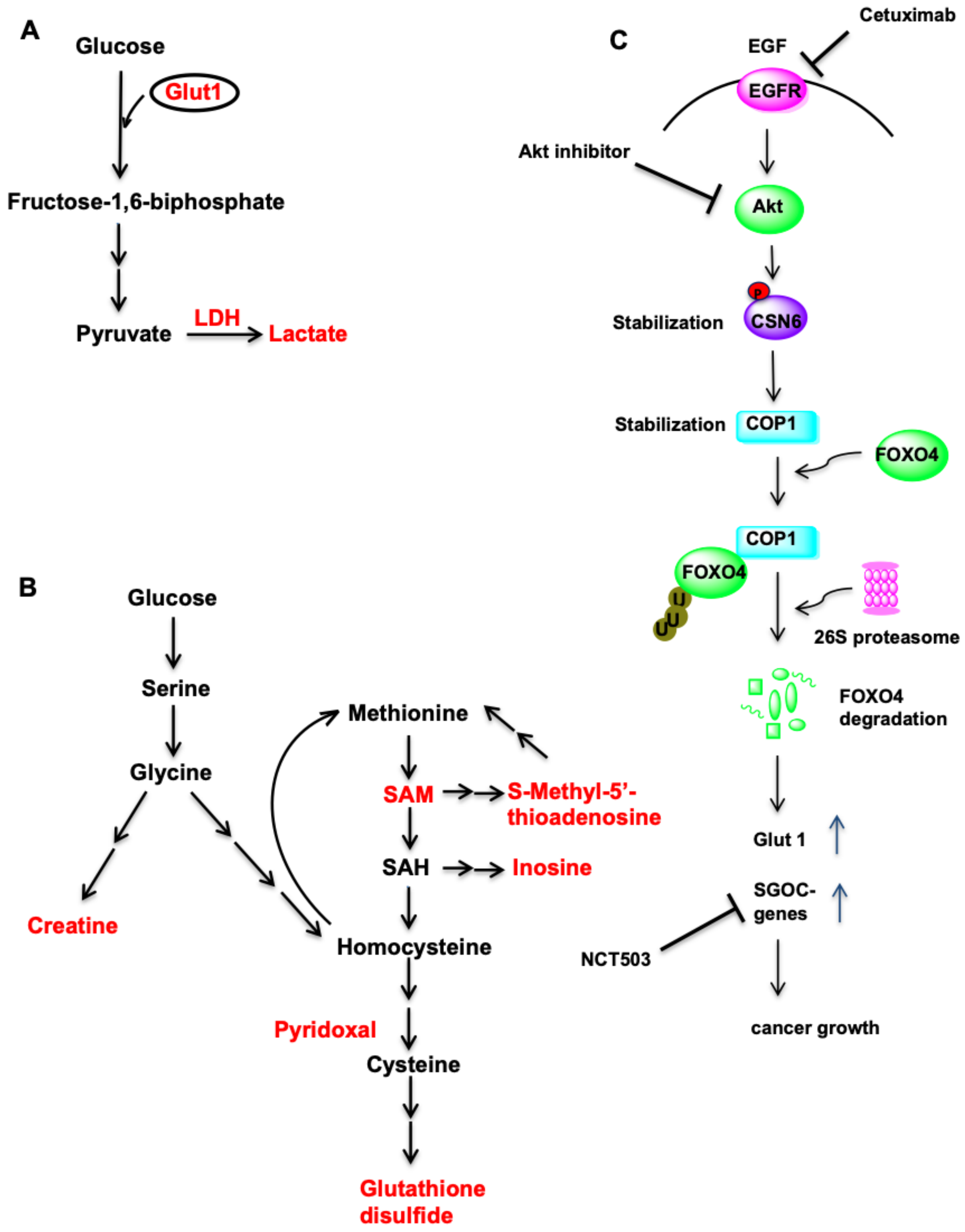

Supplementary Figure 21.

Regulation of CSN6-COP1-FOXO4 axis. (**A-B**) Pathways of metabolites that are regulated in this study. (**C**) Model of EGF signaling in regulating CSN6 and FOXO activity to promote cancer growth. Inhibitors that can be employed to intercept these regulations are indicated.

Supplementary Table 1. Correlation between expression of CSN6-FOXO4 axis and clinicopathological features of colorectal cancer patients

	CSN6 expression			FOXO4 expression		
Variable	Low(%)	High(%)	p value[a]	Low(%)	High(%)	p value[a]
Gender			0.439			0.510
Male	58(54.7)	68(49.3)		77(50.0)	49(54.4)	
Female	48(45.3)	70(50.7)		77(50.0)	41(45.6)	
age			0.605			0.894
<59 years	52(49.1)	62(44.9)		71(46.1)	43(47.8)	
≥59 years	54(50.9)	76(55.1)		83(53.9)	47(52.2)	
Histological grade			0.884			0.172
G1	7(6.6)	11(8.0)		8(5.2)	10(11.1)	
G2	82(77.4)	107(77.5)		120(77.9)	69(76.7)	
G3	17(16.0)	20(14.5)		26(16.6)	11(12.2)	
pT status			0.109			0.193
T1	6(5.7)	2(1.4)		3(1.9)	5(5.6)	
T2	18(17.0)	15(10.9)		20(13.0)	13(14.4)	
T3	81(76.4)	118(85.5)		127(82.5)	72(80.0)	
T4	1(0.9)	3(2.2)		4(2.6)	0(0)	
pN status			0.141			0.273

N0	61(57.5)	93(67.4)		93(60.4)	61(67.8)	
N1	45(42.5)	45(32.6)		61(39.6)	29(32.2)	
pM status			0.075			0.023
M0	98(92.5)	117(84.8)		130(84.4)	85(94.4)	
M1	8(7.5)	21(15.2)		24(15.6)	5(5.6)	
Clinical stage			0.009			0.090
I	18(17.0)	11(8.0)		16(10.4)	13(14.4)	
II	39(36.8)	68(49.3)		63(40.9)	44(48.9)	
III	41(38.7)	38(27.5)		51(33.1)	28(31.1)	
IV	8(7.5)	21(15.2)		24(15.6)	5(5.6)	

NOTE: All data are no. of patients (%).

[a]p values were calculated in SPSS16.0 using a chi-square test. p values <0.05 were considered to indicate statistical significance.
